# Quantitative Phosphoproteomic Analysis Reveals the Regulatory Networks of *Elovl6* on Lipid and Glucose Metabolism in Zebrafish

**DOI:** 10.3390/ijms21082860

**Published:** 2020-04-19

**Authors:** Xueting Wang, Shouxiang Sun, Xiaojuan Cao, Jian Gao

**Affiliations:** 1 College of Fisheries, Engineering Research Center of Green development for Conventional Aquatic Biological Industry in the Yangtze River Economic Belt, Ministry of Education/National Demonstration Center for Experimental Aquaculture Education, Huazhong Agricultural University, Wuhan 430070, China; xueting113@webmail.hzau.edu.cn (X.W.); ssx0707@webmail.hzau.edu.cn (S.S.); caoxiaojuan@mail.hzau.edu.cn (X.C.); 2 College of Fisheries, Hubei Provincial Engineering Laboratory for Pond Aquaculture, Huazhong Agricultural University, Wuhan 430070, China

**Keywords:** zebrafish, *elovl6* knockout, transcriptomics, proteomics, phosphoproteomics, lipid/glucose metabolism

## Abstract

Elongation of very long-chain fatty acids protein 6 (Elovl6) has been reported to be associated with clinical treatments of a variety of metabolic diseases. However, there is no systematic and comprehensive study to reveal the regulatory role of Elovl6 in mRNA, protein and phosphorylation levels. We established the first knock-out (KO), *elovl6^−/−^*, in zebrafish. Compared with wild type (WT) zebrafish, KO presented significant higher whole-body lipid content and lower content of fasting blood glucose. We utilized RNA-Seq, tandem mass tag (TMT) labeling-based quantitative technology and liquid chromatography-tandem mass spectrometry (LC-MS/MS) to perform the transcriptomic, proteomic and phosphoproteomic analyses of livers from WT and *elovl6^−/−^* zebrafish. There were 734 differentially expressed genes (DEG) and 559 differentially expressed proteins (DEP) between *elovl6^−/−^* and WT zebrafish, identified out of quantifiable 47251 transcripts and 5525 proteins. Meanwhile, 680 differentially expressed phosphoproteins (DEPP) with 1054 sites were found out of quantifiable 1230 proteins with 3604 sites. Gene ontology (GO) and kyoto encyclopedia of genes and genomes (KEGG) analysis of the transcriptomic and proteomic data further suggested that the abnormal lipid metabolism and glucose metabolism in KO were mainly related to fatty acid degradation and biosynthesis, glycolysis/gluconeogenesis and PPAR signaling pathway. Based on phosphoproteomic analyses, some kinases critical for lipid metabolism and glucose metabolism, including ribosomal protein S6 kinase (Rps6kb), mitogen-activated protein kinase14 (Mapk14) and V-akt murine thymoma viral oncogene homolog 2-like (Akt2l), were identified. These results allowed us to catch on the regulatory networks of *elovl6* on lipid and glucose metabolism in zebrafish. To our knowledge, this is the first multi-omic study of zebrafish lacking *elovl6*, which provides strong datasets to better understand many lipid/glucose metabolic risks posed to human health.

## 1. Introduction

Lipid metabolism is a complex physiological process for organisms. Normal lipid metabolism is essential for maintaining health status, as lipids participate in many biologic processes such as nutrition regulation and homeostasis [1]. Moreover, lipid metabolism disorder is the primary character of many metabolic diseases, such as fatty liver, non-alcoholic fatty liver disease (NAFLD), insulin resistance, type 2 diabetes (T2D), atherosclerosis, cancers and obesity [2,3,4,5,6,7]. In addition, many abnormalities in lipid metabolism affect glucose metabolism [8,9]. The prevalence of metabolic diseases has shown a sharp increase in the past two decades; it is urgent to develop new ways to treat these diseases [10].

Elongation of very long-chain fatty acids protein 6 (Elovl6), a member of very long-chain fatty acid elongation family, is one of the key lipogenic enzymes and regulates fatty acid metabolism in animals [11]. It is most highly expressed in the liver and mainly catalyzes palmitate (C16:0) and palmitoleate (C16:1n-7) to stearate (C18:0) and oleate (C18:1n-9), respectively [12,13]. Elovl6 can be regulated by transcription factors, such as sterol regulatory element-binding protein 1 (*Srebp-1*) [14] and carbohydrate response element binding protein (ChREBP), a mediator of glucose-induced gene expression [15]. A plenty of studies indicate that Elovl6 is an important inducible factor of many metabolic diseases [16]. It has demonstrated that the loss of *Elovl6* could reduce the hepatic injury induced by low-density lipoprotein receptor (*Ldlr*)-deficiency in the lithogenic diet-fed mice [17]. Overexpression of hepatic ELOVL6 in mice could increase hepatic inflammation, fibrogenesis and injury [18]. Takamura et al. [19] found that the cholesterol content in *Elovl6^−/−^* mice was significantly reduced, indicating that the knockout of *Elovl6* can increase cholesterol consumption and inhibit lipid accumulation. After knocking out *Elovl6* in mice, β-cell mass increased significantly and insulin adaptability increased, which improved blood glucose control [20]. The *Elovl6^−/−^* mice showed obesity and liver fat deposition, but at the same time they were protected against the high-fat and high-sucrose (HF-HS) diet induced insulin resistance [13]. Although it has been proved that ELOVL6 is a key enzyme in intracellular lipid metabolism and is closely associated with fatty liver and diabetes [21], there are no systematic and comprehensive researches of the effects of Elovl6 in lipid metabolism and glucose metabolism.

With the rapid development of high-throughput-screening technology (HT), the omics techniques which can screen a large number of genes or proteins, gain popularity so that people can systematically understand the correlativity of molecular components [22,23]. There are many regulated processes during protein synthesis, such as protein phosphorylation, an important post-translational modification regulating transcription, protein function, interactions of proteins and signal transduction [24,25]. Previously, Gassaway et al. [26] investigated the role of PKCε in lipid-induced hepatic insulin resistance by phosphoproteomic analysis, expanding the potential therapeutic targets for insulin resistance and diabetes. Matsuzaka et al. [13] reported that the knockout of *Elovl6* affected phosphorylation levels of certain kinases, thus influencing metabolism. Therefore, the application of phosphoproteomic analysis would be a good opportunity to comprehensively and systematically study the precise molecular mechanisms of Elovl6.

Zebrafish, as a model animal, have high genetic homology and several similar organ systems to humans [27]. We here first generated *elovl6^−/−^* zebrafish (KO) by CRISPR/Cas9 technique and then utilized RNA-Seq, TMT labeling-based quantitative technology and liquid chromatography-tandem mass spectrometry (LC-MS/MS) to perform comparative transcriptomic, proteomic and phosphoproteomic analyses of liver tissues between wild type zebrafish (WT) and KO zebrafish. This study aimed to identify differentially expressed genes (DEG), proteins (DEP), phosphoproteins (DEPP) and phosphosites in *elovl6^−/−^* zebrafish in comparison to WT and to further investigate the highly enriched pathways so that we can provide a comprehensive and systematic insight into the regulatory networks of *elovl6*. Meanwhile, our results also provide the potential therapeutic targets for *elovl6*-involved metabolic diseases risking to human health.

## 2. Results

### 2.1. Generation of Elovl6^−/−^ Zebrafish and Changes in Lipid/Glucose Metabolic Features between Elovl6^−/−^ and WT Zebrafish

**Figure 1:**
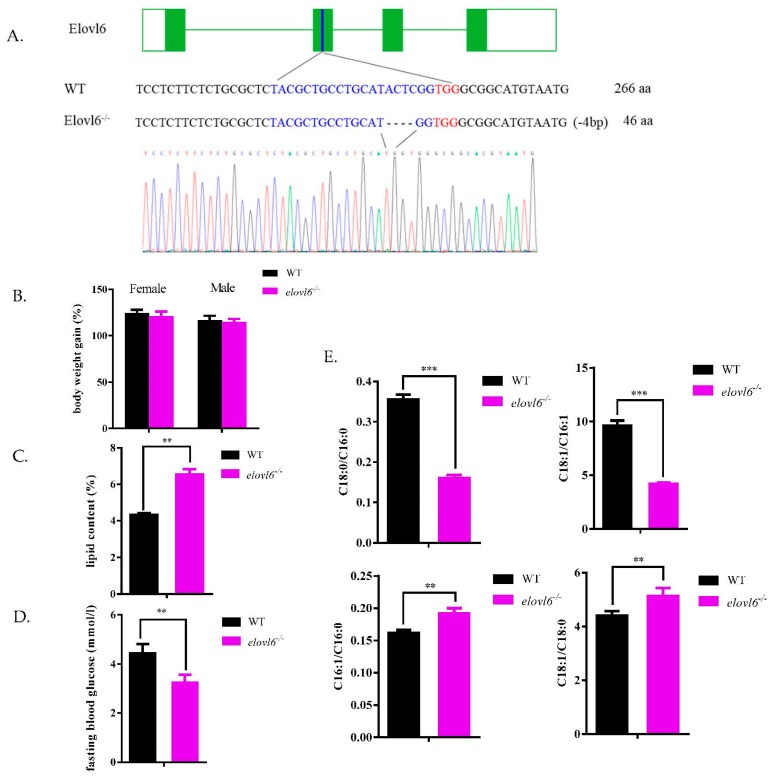
Targeting of the *elovl6* and changes in lipid/glucose metabolic features between wild type zebrafish (WT) and *elovl6^−/−^* zebrafish. (**A**) Targeting of the *elovl6* in WT and *elovl6^−/−^* zebrafish. The obtained *elovl6^−/−^* zebrafish lacked 4 bp bases, causing protein translation to stop prematurely. Introns were green lines, exons green squares and the target site blue square. (**B**) Body weight gains of WT and *elovl6^−/−^* zebrafish females/males (the ratio of the weight of two-month-old fish to the weight of one-month-old fish,%; *n* = 4). (**C**) The whole-body lipid contents of WT and *elovl6^−/−^* zebrafish (*n* = 4). (**D**) Fasting blood glucose contents of WT and *elovl6^−/−^* zebrafish (*n* = 3). (**E**) The ratios of C18:0 to C16:0, C18:1 to C16:1, 18:1 to 18:0 and 16:1 to 16:0 in whole bodies of WT and *elovl6^−/−^* zebrafish (*n* = 4). Results are represented as means ± SD. ** *p* < 0.01, *** *p* < 0.001. *elovl6*, elongation of very long chain fatty acids protein 6.

To define the specific roles of Elovl6 in zebrafish, we first generated *elovl6^−/−^* zebrafish by CRISPR/Cas9 technique. We disrupted the 2nd exon of *elovl6* and generated *elovl6*-specific mutations with “ACTC” deletion determined by PCR and sequencing, which led to premature stop and shorten the length of the original protein (266 amino acids) to 46 amino acids (Figure 1A). We found that the *elovl6^−/−^* zebrafish presented significantly lower hepatic *elovl6* mRNA level than WT zebrafish (Figure S1A). There were no significant differences in body weight gains of females and males between WT and *elovl6^−/−^* zebrafish (Figure 1B). The whole-body lipid content significantly increased in *elovl6^−/−^* zebrafish in comparison to WT (Figure 1C). The fasting blood glucose contents in WT and *elovl6^−/−^* zebrafish were evaluated. As shown in Figure 1D, the blood glucose level of *elovl6*-deletion zebrafish was significantly lower than that of WT fish. By analyzing the fatty acid composition of whole fish (Table S1), we found that the C18/C16 ratio of *elovl6^−/−^* zebrafish decreased significantly, compared with that of WT zebrafish (Figure 1E). It indicated that the function of elongating C16 to C18 was impaired in *elovl6^−/−^* zebrafish. At the same time, significant increases in the ratios of C16:1/C16:0 and C18:1/C18:0 were found in *elovl6^−/−^* zebrafish in comparison to WT, indicating that the desaturation of *elovl6*-deletion zebrafish was enhanced (Figure 1E).

### 2.2. Comparative Analysis of Transcriptomics between Elovl6^−/−^ and WT Zebrafish

**Figure 2:**
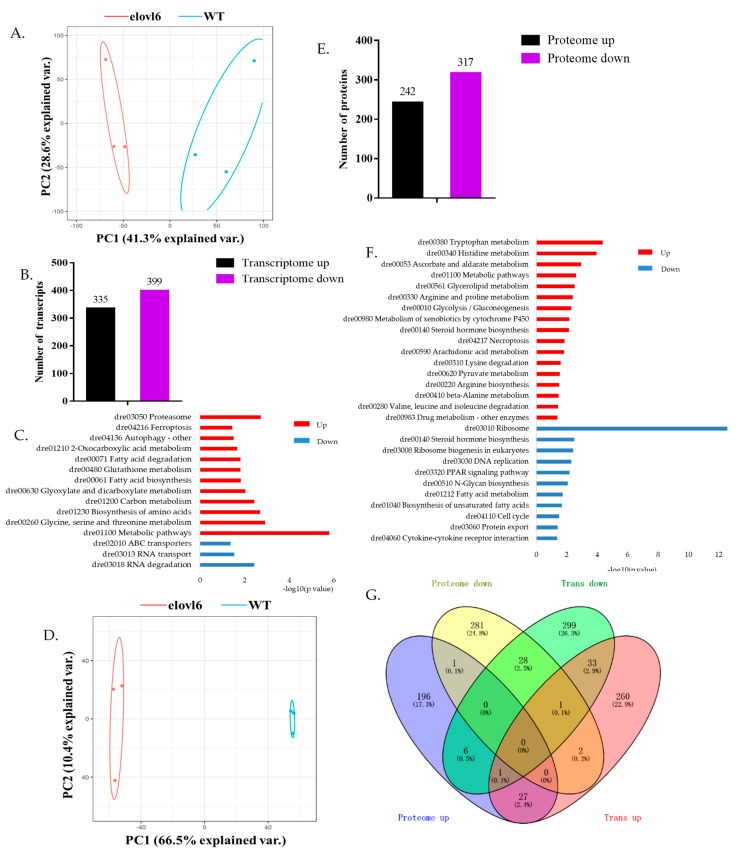
Comparative analyses of hepatic transcriptomics and proteomics between *elovl6^−/−^* and WT zebrafish and the integrated analysis of transcriptomics and proteomics. (**A**) The principal component analysis (PCA) at the transcriptomic level for a total of six samples of the *elovl6^−/−^* and wild type (WT) zebrafish. (**B**) Up- and down-regulated DEG in *elovl6^−/−^* zebrafish in comparison to WT. (**C**) Analysis of KEGG pathway enrichment of DEG to understand the biologic processes. (**D**) The principal component analysis (PCA) at the proteomic level for a total of six samples of the *elovl6^−/−^* and WT zebrafish. (**E**) Up- and down-regulated DEP in *elovl6^−/−^* zebrafish in comparison to WT. (**F**) Analysis of KEGG pathway enrichment of DEP to understand the biologic processes. (**G**) Overlap between DEP and DEG. DEG, differentially expressed genes; DEP, differentially expressed proteins; *elovl6*, elongation of very long chain fatty acids protein 6; KEGG, Kyoto Encyclopedia of Genes and Genomes; *elovl6*, elongation of very long chain fatty acids protein 6.

The hepatic transcriptomic data of *elovl6^−/−^* and WT zebrafish were analyzed to evaluate the quantitative repeatability using the principal component analysis (PCA) and the corresponding two-dimensional scatter plot showed high repeatability (Figure 2A). There were 734 DEG identified; 335 of them were up-regulated and 399 down-regulated in *elovl6^−/−^* zebrafish in comparison to WT (Figure 2B). Moreover, the Kyoto Encyclopedia Of Genes and Genomes (KEGG) database was used to perform the pathways analysis for the functional enrichment of DEG. Figure 2C shows top 15 KEGG pathways enriched by DEG, including 12 up-regulated and three down-regulated pathways in *elovl6^−/−^* zebrafish. The metabolic pathway was the most enriched up-regulated pathway; RNA degradation the most enriched down-regulated pathway. In addition, many DEG were involved in insulin signaling pathway, peroxisome proliferators-activated receptor (PPAR) signaling pathway and glycolysis/gluconeogenesis (Table S2). We randomly selected 10 genes (acyl-CoA synthetase bubblegum family member 2 (*acsbg2*), fatty acid synthase (*fasn*), phosphotransferase (*gck*), phosphoenolpyruvate carboxykinase 2 (mitochondrial) (*pck2*), solute carrier family 27 member 1b (*slc27a1b*), glycogen synthase 2 (liver) (*gys2*), hexokinase domain-containing 1 (*hkdc1*), lipoprotein lipase (*lpl*), mitochondrial uncoupling protein 1 (*ucp1*) and *ucp2*) for qPCR verification to confirm the RNA-Seq results. The results of qPCR verification of 10 genes showed the same gene expression patterns as RNA-seq results (Figure S2).

### 2.3. Identification of DEP between Elovl6^−/−^ and WT Zebrafish

The main peptides were 7–20 amino acids in length, which conformed to the general rules based on trypsin digestion and high energy collision-induced dissociation (HCD) fragmentation (Figure S3A). The data were analyzed to evaluate the quantitative repeatability using PCA and the corresponding two-dimensional scatter plot showed high repeatability (Figure 2D). A total of 5525 quantifiable proteins in liver tissues of zebrafish were identified (Table S3). There were 559 DEP, including 242 up-regulated and 317 down-regulated DEP in *elovl6^−/−^* zebrafish in comparison to WT (Figure 2E and Table S3). Figure 2F shows top 30 KEGG pathways enriched by DEP, including 18 up-regulated and 12 down-regulated pathways in *elovl6^−/−^* zebrafish in comparison to WT. Many pathways were related to glucose metabolism and lipid metabolism, such as steroid hormone biosynthesis metabolism, glycolysis/gluconeogenesis, pentose and glucuronate interconversions, glycerolipid metabolism, pyruvate metabolism, arachidonic acid metabolism, PPAR signaling pathway, biosynthesis of unsaturated fatty acids and fatty acid metabolism. These results indicated that the Elovl6 may regulate glucose metabolism and lipid metabolism through these pathways. Further, we summarized the DEP related to the pathways we were interested in: insulin signaling pathway, PPAR signaling pathway, glycolysis/gluconeogenesis and fatty acid metabolism (Table S4). Among them, phosphoenolpyruvate carboxykinase 1 (Pck1) involved in three pathways: insulin signaling pathway, PPAR signaling pathway and glycolysis/gluconeogenesis. Stearoyl-CoA desaturase (Scd), Acyl-CoA synthetase long chain family member 1b (Acsl1b) and Acsl5 involved in two pathways, namely PPAR signaling pathway and fatty acid metabolism. These four DEPs were all down-regulated in *elovl6^−/−^* zebrafish in comparison to WT.

### 2.4. Integrated Analysis of Transcriptomics and Proteomics

There were 5540 genes identified in the transcriptome and proteome data. We found that 1135 of these genes were significant differentially expressed between *elovl6^−/−^* and WT zebrafish, including 555 up-regulated and 652 down-regulated genes in *elovl6^−/−^* zebrafish (Figure 2G). The 1135 genes can be divided into eight different expression types (TrUp, TrDown, PrUp, PrDown, TrUpPrUp, TrDownPrDown, TrUpPrDown and TrDownPrUp) and each type can correspond to a regulatory relationship. Further, the KEGG enrichment analysis of these genes was performed (Figure S4). Nine KEGG pathways for PrUpTrUp and two KEGG pathways for PrDownTrDown were found. In addition, there were 28 genes (5.0%) up-regulated and 29 genes (4.4%) down-regulated at both transcriptomic and proteomic datasets (Figure 2G), indicating that these genes may be closely related to *elovl6*. Among these genes, we found many genes related to lipid metabolism and glucose metabolism, such as *ldlra*, L-lactate dehydrogenase B-B chain (*ldhbb*) and hydroxy-delta-5-steroid dehydrogenase, 3 beta-and steroid delta-isomerase (*hsd3b7*).

### 2.5. Global identification of DEPP between elovl6^−/−^ and WT zebrafish

**Figure 3:**
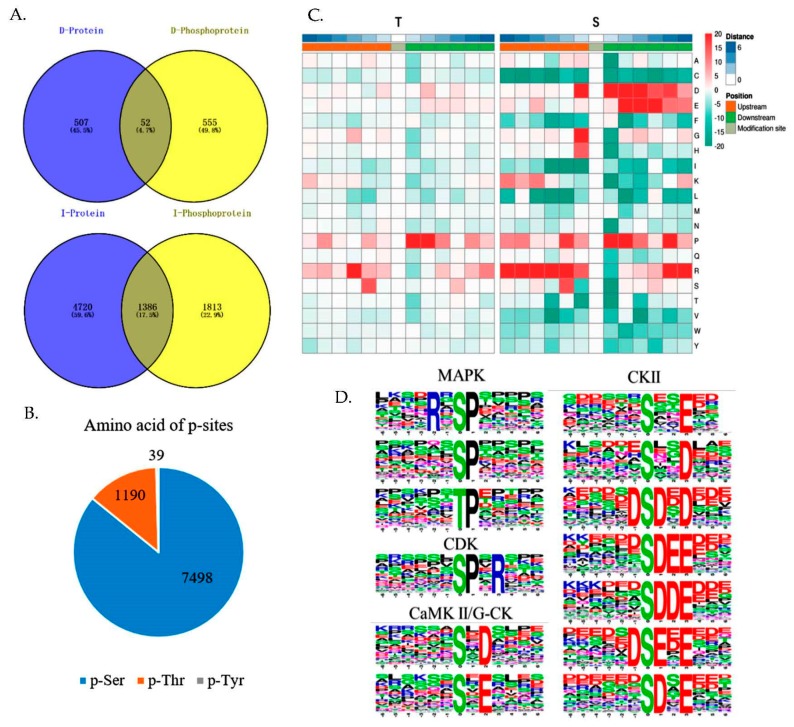
The overlaps of the proteomic and phosphoproteomic data and motif analysis of the identified phosphosites. (**A**) The overlap of differentially expressed proteins and phosphoproteins (top) and overlap of identified proteins and phosphoproteins (bottom). (**B**) Distribution of the number of different amino acid modification sites identified. (**C**) The motif enrichment heatmap of up-stream and down-stream amino acids of all identified modification sites. Red indicates that the amino acid is significantly enriched near the modification site and green significantly reduced near the modification site. (**D**) Significantly enriched phosphorylation motifs (CKII, MAPK, CDK, CaMK II/G-CK) of *elovl6^−/−^* zebrafish vs. wild type zebrafish. D-Protein/Phosphoprotein, differentially expressed proteins/phosphoproteins; I- Protein/Phosphoprotein, identified proteins/phosphoproteins; CaMK II, calcium/calmodulin kinase II; CDK, cyclin-dependent kinase; CKII, casein kinase II; *elovl6*, elongation of very long-chain fatty acids family member 6; G-CK, Golgi casein kinase; MAPK, mitogen-activated protein kinase.

The lengths of most peptides were conformed with the general rules of trypsin digestion and HCD fragmentation, indicating the results were reliable (Figure S3B). In total, there were 3199/1230 phosphoproteins identified/quantified and 8727/3604 phosphosites identified/quantified (Figure S5 and Table S3). The phosphoproteins and phosphosites with the fold change ≥1.5 (or fold change ≤0.66) and *p*-value ≤0.05 were identified as DEPP and differentially expressed phosphosites. As a result, there were 680 DEPP, including 224 up-regulated and 456 down-regulated DEPP in *elovl6^−/−^* zebrafish in comparison to WT and 1054 differentially expressed phosphosites, including 289 up-regulated and 765 down-regulated phosphosites in the *elovl6^−/−^* zebrafish relative to WT (Table S3). By comparing the data of proteomics and phosphoproteomics, we found 1813 (56.7%) identified phosphoproteins and 555 (91.4%) DEPP were not present in the proteome dataset (Figure 3A). Most identified phosphosites were found to be singly and doubly and just a few phosphosites were multiply (Figure S6). Meanwhile, 7498 (85.9%), 1190 (13.6%) and 39 (0.5%) phosphosites were at serine (p-Ser), threonine (p-Thr) and tyrosine (p-Tyr), respectively (Figure 3B). In addition, we summarized the phosphorylation level changes in DEPP related to insulin signaling pathway, glycolysis/gluconeogenesis and lipid metabolism (Table S5).

### 2.6. Motif Analysis of Identified p-Sites

The motif analysis of protein was based on the statistics of the amino acid sequences before and after phosphorylation sites to summarize the regularity of amino acid sequences in the regions of phosphorylation sites, inferring the enzymes related to modifications. Figure 3C shows the heat map of motif enrichment of amino acid upstream and downstream of p-Ser and p-Thr. In the motif enrichment heat map, proline (P) residues had high frequency in the downstream of the p-Ser and p-Thr and arginine (R) residues, aspartic acid (D) and glutamic acid (E) residues only had high frequency in the upstream or downstream of the p-Ser. The presences of R at upstream 3 and S at upstream 2 were determined around the p-Ser and p-Thr. The motif-x analysis showed that 61 phosphorylation motifs were enriched in the phosphoproteome of *elovl6^−/−^* vs. WT (Table S6). Further, based on previous researches [28,29], we found that casein kinase II (CKII) recognized the most motifs, including: xxxxxx_S_xxExxx, xxxxxx_S_xxDxxx, xxxxxD_S_DxDxxx, xxxxxx_S_DEExxx, xxxxxx_S_DDExxx, xxxxxD_S_ExExxx, xxxxxx_S_DxExxx. There were motifs recognizable by mitogen-activated protein kinase (MAPK): xxxxPx_S_Pxxxxx, xxxxxx_S_Pxxxxx, xxxxxx_T_Pxxxxx. Motifs recognized by cyclin-dependent kinase (CDK): xxxxxx_S_PxRxxx and motifs by calcium/calmodulin kinase II (CaMK II) and Golgi casein kinase (G-CK): xxxxxx_S_xDxxxx, xxxxxx_S_xExxxx, were found (Figure 3D). These results suggested that these motifs may be important for phosphorylation modification.

### 2.7. Functional Analysis of DEP and DEPP

**Figure 4:**
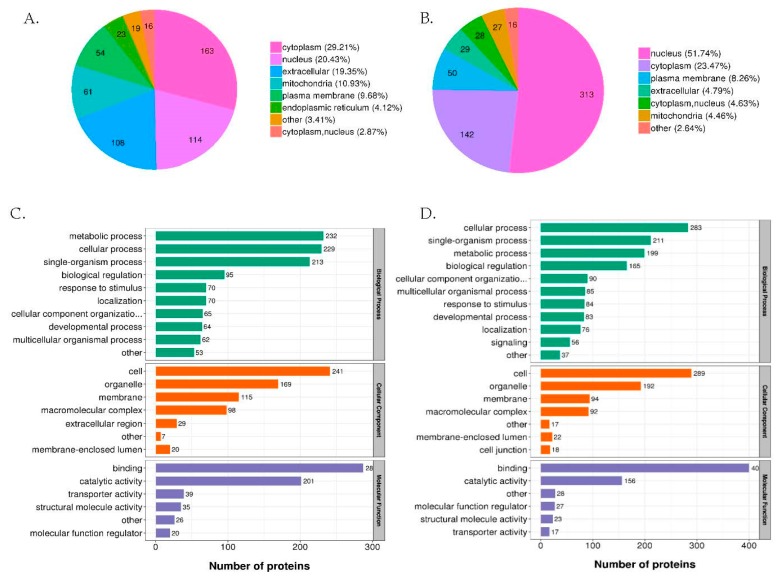
Functional analysis of differentially expressed proteins (DEP) and phosphoproteins (DEPP) between wild type (WT) and *elovl6^−/−^* zebrafish. A and B. Subcellular localization charts of DEP (**A**) and DEPP (**B**). C and D. The column diagrams show GO (2nd Level) enrichment of DEP (**C**) and DEPP (**D**) in three categories (biologic process, cellular component and molecular function). *elovl6*, elongation of very long chain fatty acids protein 6; GO, gene ontology.

We used the software WoLF PSORT to predict the subcellular localization of the DEP and DEPP. Subcellular localization plays an important role in predicting and identifying protein function; studying protein function by subcellular localization is one of the most critical research contents in proteomics analysis [30]. In proteomics analysis, DEP were most abundant in the cytoplasm (29.21%), followed by the nucleus (20.43%) and in the phosphoproteomics, the DEPP were mainly in the nucleus (51.74%), followed by the cytoplasm (23.47%) (Figure 4A,B). These results indicated that the DEP and DEPP were mainly in the cytoplasm and nucleus, which were the main locations for the intracellular activity [31].

Gene ontology (Go) analysis of proteomics showed that the metabolic process, cell and binding were the main classifications of three categories (biologic process (BP), cellular compartment (CC), molecular function (MF)) in the GO category (2^nd^ level). However, for phosphoproteomics, the main classification in BP category was the cellular process (Figure 4C,D). Moreover, the results of GO enrichment revealed more specific functional enrichment of DEP and DEPP (Figure S7A,B). There was something noticeable that down-regulated DEP in the proteomics were mainly enriched in molecular function of lipid transport, regulation of lipase activity, positive regulation of lipase activity and steroid biosynthetic process and biologic processes of lipid transporter activity. While in the phosphoproteomics, the main GO enrichment of DEPP were related to the molecular function of phosphatase regulator activity, protein phosphatase regulator activity, protein phosphatase inhibitor activity and phosphatase inhibitor activity and biologic processes of cell metabolism. Taken together, the results of GO enrichment analysis showed that the *elovl6* deletion mainly caused changes in the abundance of proteins related to lipid metabolism and changes in the phosphorylation level of proteins related to the activity of phosphatase regulators.

### 2.8. Protein-Protein Interaction Networks of DEPP

**Figure 5:**
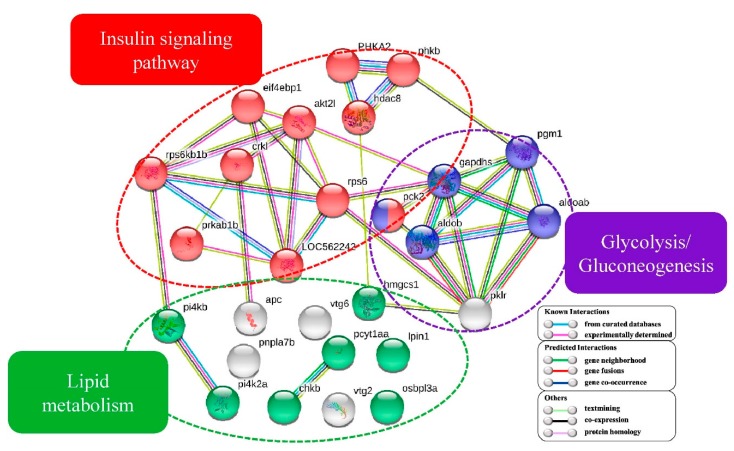
Protein-protein interaction networks of twenty-seven differentially expressed phosphoproteins (DEPP) associated with lipid metabolism and glucose metabolism. Akt2l, V-akt murine thymoma viral oncogene homolog 2, -like; Aldoab, fructose-bisphosphate aldolase; Aldob, fructose-bisphosphate aldolase B; Crkl, V-crk avian sarcoma virus CT10 oncogene homolog-like; Eif4ebp1, eukaryotic translation initiation factor 4E-binding protein 1; Gapdhs, glyceraldehyde-3-phosphate dehydrogenase; Hdac8, phosphorylase b kinase regulatory subunit; Pck2, phosphoenolpyruvate carboxykinase 2 (mitochondrial); pgm1, phosphoglucomutase-1; Pklr, pyruvate kinase; Phka2, phosphorylase b kinase regulatory subunit; Prkab1b, protein kinase, AMP-activated, beta 1 non-catalytic subunit, b; Rps6kb, ribosomal protein S6 kinase b 1b; Rps6, 40S ribosomal protein S6; Apc, adenomatous polyposis coli; Osbpl3a, oxysterol-binding protein; Pnpla7b, patatin-like phospholipase domain-containing 7b; Pi4kb, phosphatidylinositol 4-kinase beta; Pi4k2a, phosphatidylinositol 4-kinase type 2-alpha; Lpin1, lipin 1; Pcyt1aa, phosphate cytidylyltransferase 1, choline, alpha a; Hmgcs1, 3-hydroxy-3-methylglutaryl coenzyme A synthase; Vtg6, vitellogenin 6; Vtg2, vitellogenin 2; Chkb, choline kinase beta.

Protein-protein interactions (PPI) have attracted attention and are used to better understand cellular mechanisms. Here, we applied the software STRING11.0 to construct protein-protein interaction networks by submitting DEPP related to insulin signaling pathway, glycolysis/glycogenolysis and lipid metabolism. Among them, Pck2 was involved in both the insulin signaling pathway and glycolysis/gluconeogenesis pathway (Figure 5).

### 2.9. Interaction Analysis of Differentially Expressed Kinases

Kinases are enzymes that catalyze the phosphorylation of substrates and play an important role in organism metabolism. Here, 28 differentially expressed and differentially modified kinases were submitted to conduct blast searching against the existing databases in STRING 11.0 software (Table S7). According to the number of involved kinases, we selected the top five signaling pathways. They were insulin signaling pathway (7 kinases participated in), MAPK signaling pathway (6 kinases participated in), mammalian target of rapamycin (mTOR) signaling pathway (4 kinases participated in), gonadotropin-releasing hormone (GnRH) signaling pathway (4 kinases participated in) and apelin signaling pathway (4 kinases participated in). The highest frequency of these kinases enriched in these pathways was Akt21, followed by Rps6kb1b, Rps6kb1a, Rps6ka3a, Mapk14b, mitogen-activated protein kinase 6 (Map2k6) and protein kinase, AMP-activated, beta 1 non-catalytic subunit, b (Prkab1b). Among them, the phosphorylation levels of Akt21 and Prkab1b increased and Mapk14b, Rps6ka3a, Rps6kb1a and Rps6kb1b decreased in *elovl6^−/−^* zebrafish in comparison to WT zebrafish.

## 3. Discussion

In this study, we took *elovl6* deletion zebrafish as the subject to reveal the regulatory networks of *elovl6*, which is a potential therapeutic target for many lipid/glucose metabolic diseases. The zebrafish lacking *elovl6* showed lower ratios of C18:0/C16:0 and C18:1/C16:1, indicating the extending function of Elovl6 (catalyzing C16 to C18) was impaired in *elovl6^−/−^* zebrafish. Similarly, the decreased ratio of C18:0/C16:0 was also found in *elovl6^−/−^* mice [13]. Our results revealed the critical role of ELOVL6 in fatty acid elongation, and strongly suggested a conserved architecture between teleosts and mammals [32]. Following this, we applied the *elovl6^−/−^* zebrafish model and WT zebrafish to identify their DEG, DEP and DEPP and explored the precise molecular mechanisms of *elovl6* in lipid/glucose metabolism.

Interestingly, 10 DEPs belonged to the PPAR signaling pathway were found between WT and *elovl6^−/−^* zebrafish. PAPRs are nuclear hormone receptors that are activated by fatty acids and their derivatives. The enzyme Scd1 is the rate-limiting enzyme for oleic acid biosynthesis; it has recently been shown to be the critical control point regulating hepatic lipogenesis and lipid oxidation [33]. Our results showed that zebrafish lacking *elovl6* suppressed the expression level of Scd in liver. It suggests loss of *elovl6* may reduce lipogenesis in zebrafish. Similar results were found in other studies [20,34]. The expression contents of the key enzymes related to lipid transport including Acsl1 and Acsl5 significantly decreased. Acsl1 and Acsl5 are well documented for their roles in complex fatty acid breakdown, fatty acid channeling and transport of fatty acids to mitochondria for their oxidation [35,36]. Decreased protein levels of these two enzymes could suggest that ablation of *elovl6* impaired fatty acid β-oxidation. Taken together, these could explain why zebrafish lacking *elovl6* reduced lipogenesis but the whole-body lipid content increased. The reduced fatty acid β-oxidation could be effective at increasing the accumulated lipid in body. Meanwhile, we identified genes related to lipid and glucose metabolism at both the transcript and protein levels, including *ldlra, ldhbb* and *hsd3b7*. Previously, knockout of *Ldlr* led to severe dyslipidemia and liver steatosis, and the absence of *Elovl6* can relatively reduce the symptoms [17]. LDHB catalyzes the conversion of lactate to pyruvate, which plays a key role in glycolysis/gluconeogenesis [37]. Hsd3b7, a rate-limiting enzyme, plays an important role in the lipid (cholesterol) metabolism in liver tissue [38]. Our results indicated that the combination of the proteome and transcriptome can bring more reliable information than that from the proteome or transcriptome alone.

Phosphorylated proteins are important post-translational modifications that regulate various physiological functions, and the level of phosphorylation is mainly regulated by protein kinases [39]. Previous studies have shown that MAPK14 is involved in energy metabolism in hepatocytes [40]. Meanwhile, it was also found that both medium-chain and long-chain fatty acids can activate and phosphorylate MAPK14, thereby inducing the expression of gluconeogenesis genes in liver cells [41]. In this study, knocking out *elovl6* in zebrafish prevented the elongation of fatty acid chains, and the results of phosphoproteomic analysis also showed that Mapk14b phosphorylation level was reduced. Meanwhile, *elovl6*-knockout zebrafish has lower blood glucose compared with WT, which indicated that gluconeogenesis may be weakened in *elovl6^−/−^* zebrafish. Therefore, down-regulated phosphorylation of Mapk14b in *elovl6^−/−^* zebrafish may be related to regulating glucose metabolism.

Moreover, RPS6KB1 (p70S6) is highly expressed and phosphorylated in tumor cells [42]. The phosphorylation level of RPS6KB1 was decreased by using inhibitors, which could greatly reduce the volume of tumor cells [43]. In addition, glucose and leucine increased mTOR/p70S6 kinase phosphorylation and caused insulin resistance, while rapamycin inhibited the changes of phosphorylation and insulin resistance [44]. Furthermore, it has been demonstrated that RPS6KB1 is ribosomal protein S6 kinase, essential for protein translation and has an important role in a variety of metabolic diseases, such as obesity and diabetes [45,46,47]. In this study, the degree of phosphorylation of Rps6kb1 was found to be decreased in *elovl6^−/−^* zebrafish. Therefore, to deeply study the relationship between Elovl6 and Rps6kb1 would be a good research point.

In addition, AKT2, the major marker for insulin signaling, is one of the three isoforms of AKT [48]. It was found that the loss of *Akt2* in mice can cause insulin resistance, which leads to diabetes mellitus-like. The increase of AKT2 phosphorylation may increase the insulin sensitivity of skeletal muscle in the rat [49,50]. Previously, Matsuzaka et al. [13] found that restoration of hepatic AKT phosphorylation in *Elovl6^−/−^* mice protected them against insulin resistance. Compared with WT mice, the plasma glucose content of *Elovl6^−/−^* mice was significantly reduced. Our results showed that zebrafish lacking *elovl6* up-regulated phosphorylation of Akt2l and also showed lower blood glucose levels than WT zebrafish. Thus, the phosphorylation of Akt2l would be closely related to insulin resistance in *elovl6^−/−^* zebrafish.

In conclusion, *elovl6^−/−^* zebrafish presented significant higher content of whole-body lipid and lower content of fasting blood glucose than WT. The further quantitative omics analysis revealed changes in abundances of some proteins including Scd, Acsl1 and Acsl5 and changes in phosphorylation levels of the kinases including Akt2l, Rps6kb1 and Mapk14 between *elovl6^−/−^* and WT zebrafish, and all of these proteins/kinases were related to lipid and glucose metabolism. Meanwhile, we also found that some DEP and DEPP were greatly involved in some signaling pathways, such as insulin signaling pathway, PPAR signaling pathway and glycolysis/gluconeogenesis, which were related to lipid and glucose metabolism. Therefore, further studies of these DEP and DEPP would have profound significances for studying Elovl6 as a potential therapeutic target for many metabolic diseases such as type 2 diabetes, insulin resistance and fatty liver.

## 4. Materials and Methods

### 4.1. Ethical Approval

All experiments were conducted strictly under the Guidance Suggestions for the Care and Use of Laboratory Animals of Huazhong Agricultural University. This study was approved by the Committee on the Ethics of Animal Experiments of Huazhong Agricultural University (HZAUFI-2019-015, 10-JAN-2019).

### 4.2. Generation of Elovl6^−/−^ Zebrafish

WT zebrafish (AB strain) were purchased from the Institute of Hydrobiology of the Chinese Academy of Sciences, China Zebrafish Resource Center, Wuhan, China. They were maintained at 26–28℃ in the recirculation water system and the lighting condition was 14:10 h (light: dark). Zebrafish were fed with brine shrimp twice a day.

*Elovl6^−/−^* zebrafish were generated by CRISPR/Cas9 technique. The *elovl6* DNA sequences of zebrafish were obtained from National Center for Biotechnology Information Search database (NCBI) (http://www.ncbi.nlm.nih.gov/) and gRNA sequences were designed by using the CRISPR direct (https://crispr.dbcls.jp/). The synthesis and purification of *cas9* RNA and gRNA were performed based on the methods described by our previous study [51]. The oligonucleotides were synthesized by TsingKe Biological Technology (Wuhan, China) (Oligo-F: TGTAATACGACTCACTATATACGCTGCCTGCATACTCGGGTTTTAGAGCTAGAAAT; Oligo-R: AAAGCACCGACTCGGTGCCA). Then the mixture of *cas9* mRNA (1000 ng/μL) and gRNA (500 ng/μL) was microinjected into one-cell embryos of WT zebrafish. We cultured the founder (F0) embryos to adult zebrafish and heterozygous embryos (F1) were obtained by hybridizing F0 with WT zebrafish. Finally, we obtained *elovl6^−/−^* zebrafish by crossing the F1 generation. We extracted genomic DNA from the caudal fins and performed PCR to amplify target sites to find *elovl6* homozygous zebrafish (primer sequences: F: TCGGTCAGTACGGAAAATAAAT; R: TAACAGGTGCTGGAGGCAAAAA). The specific methods were as described by Mastrodonato et al. [52]. There were no morphologic defects in *elovl6^−/−^* zebrafish embryos. We measured the weights of the fish (WT and *elovl6^−/−^* zebrafish) at the ages of one month and two months and obtained the body weight gains (namely, the ratio of the weight of two-month-old fish to the weight of one-month-old fish,%).

In this study, two-month-old WT and *elovl6^−/−^* zebrafish males were used for the transcriptomic, proteomic and phosphoproteomic analyses. The corresponding workflow is shown in Figure S1B.

### 4.3. RNA Extraction and Quantitative PCR (qPCR) Analysis

Total RNA was extracted from WT and *elovl6^−/−^* zebrafish liver using Trizol (TaKaRa Bio, Inc., Tokyo, Japan) and the specific steps were carried out according to the instructions. The SmartSpec ™ Plus spectrophotometer (BioRad, Inc., Hercules, CA, USA) was used to determine the RNA concentration and purity. The RNA was reverse transcribed into cDNA by cDNA synthesis kit (Yeasen Biotech, Co., Ltd., Shanghai, China) and the specific steps and dosage were performed according to the instructions. After obtaining the cDNA, qPCR test was performed using the Mini Opticalon real-time detector (BIO-RAD, Hercules, CA, USA) to determine the expression patterns of the selected genes. The solutions in the qPCR reaction included 5 μL SYBR^®^ premix Ex Taq ™ (2×), 0.2 μL PCR forward primer (10 μM), 0.2 μL PCR reverse primer (10 μM), 1.0 μL RT reaction (5-fold diluted cDNA solution) and 3.6 μlddH2O. The gene expression level was normalized towards the mean of the two reference genes (*gapdh* and *β-Actin*), and the relative expression was calculated with the comparative Ct method (2 (^−ΔΔCt^)). The procedures of qPCR reaction were described by Zhao et al. [51]. qPCR primers are shown in Table S8.

### 4.4. Lipid Content and Fatty acid Composition Analyses

12 male WT and 12 male *elovl6^−/−^* zebrafish were used here; three individuals of each kind of zebrafish were considered as a biologic sample. The whole fish was placed in a 5 mL centrifuge tube and then 2 mL methanol/chloroform (1: 2 *v*/*v*) was added into the tube for crushing using a tissue lyser (Scientz Biotechnology Co., Ltd., Ningbo, China). Then, 1.0 mL deionized water was added and centrifuged (145× *g*, 10 min) to aspirate the lower chloroform layer and the lipid was dried with nitrogen. The total lipid content of the whole bodies of WT and *elovl6^−/−^* zebrafish were measured. The fatty acid compositions were measured by using the total lipid extracted above and performing the following operations. Methanol/sulfuric acid (100: 1*v*/*v*) with butylated hydroxytoluene (BHT) was added to methylate at 85 °C for 1.5 h and 100 °C for 30 min. After cooling to room temperature, n-hexane was added and the fatty acid methyl esters (FAME) were dried with nitrogen. The remaining supernatant was dissolved in chromatographic grade n-hexane (Sigma-Aldrich, St. Louis, MO, USA). We used a gas chromatograph (Shimadzu emit Co., Ltd., Tokyo, Japan) to determine the fatty acid compositions of whole-body WT and *elovl6^−/−^* zebrafish according to our previous methods [53]. Samples were automatically injected into the inlet. Fatty acids were calculated as a percentage of individual regions in total fatty acids.

### 4.5. Fasting Blood Glucose Measurement

Blood glucose levels were measured in fasted male fish following euthanasia in ice. Whole blood was collected from three WT/KO zebrafish [54] and then pooled into one tube as a biologic blood sample (*n* = 3). The fasting blood glucose levels were determined by a glucometer (ACCU-CHEK (Roche) glucose meter) with a test strip.

### 4.6. Transcriptomic Analysis

#### 4.6.1. RNA Isolation, Library Preparation and Sequencing

Nine WT zebrafish and nine *elovl6^−/−^* zebrafish (each kind of zebrafish three to a biologic sample) were randomly selected as experimental fish and total RNAs were extracted from the livers using Trizol (Invitrogen, CA, USA) according to the manufacturer’s instructions. Six samples in total were used for transcriptomic sequencing here. The total RNA concentration was determined by NanoDrop 2000 (Thermo Scientific, Waltham, MA) and the integrity value was checked by the RNA 6000 Pico LabChip on an Agilent 2100 Bioanalyzer (Agilent, Santa Clara, CA, USA). The purifications of total RNA and cDNA were based on methods described by Zhang et al. [55]. Thereafter, the cDNA library construction and sequencing were undertaken by MajorBio Pharmaceutical Technology (Shanghai, China). The single-end paired-end technology was used for cDNA library sequencing on the Illumina sequencing platform (Illumina HiSeq 4000 SBS Kit; Illumina, San Diego, CA, USA). The Illumina GA processing pipeline was used to analyze the image and for base calling.

#### 4.6.2. De Novo Assembly and Annotations

Prior to assembly, clean reads were obtained by filtering out adapter sequences and ambiguous nucleotides using SeqPrep (https://github.com/jstjohn/SeqPrep) and Sickle (https://github.com/najoshi/sickle). Then, all clean reads were assembled into transcripts using StringTie (http://ccb.jhu.edu/software/stringtie/), and the details were described by Zhang et al. [55]. After de novo assembly was completed, we used TGICL clustering software (J. Craig Venter Institute, Rockville, MD, USA) to cluster and remove excess transcripts, and the remaining sequences were unigenes. Then we performed the BLASTx with the E-value <10^−5^ between the unigenes, and the databases GO (http://www.geneontology.org/) and KEGG (http://www.genome.jp/kegg/) were used to annotate the transcriptome. GO annotations of these unigenes were produced using Blast2GO based on the NCBI Nr database (ftp://ftp.ncbi.nlm.nih.gov/blast/db/). We first compared these unigenes with the Nr database using BLASTn, then searched for proteins with sequences that have the highest similarity to the given unigenes and used HMMER3 to compare Pfam database (http://pfam.xfam.org/) to get the information of protein family and functional domain.

#### 4.6.3. Differentially Expressed Gene Analysis

The fragments per kilobases per million reads (FPKM) method was used to normalize mapped fragments [56]. Then, the DEG between *elovl6^−/−^* zebrafish and WT were identified by the DEG-seq software package applying the MA-plot-based method with random sampling (MARS) model methods. The *p*-value <0.005 and the absolute value of log_2_ fold change >1 were considered to have significant expression abundance. All DEG were mapped to terms in GO and KEGG database. All transcriptome data are available in the NCBI Sequence Read Archive (SRA) database under accession SRP251016.

### 4.7. Proteomic and Phosphoproteomic Analysis

#### 4.7.1. Protein Extraction and Trypsin Digestion

Protein extraction and trypsin digestion with some changes were based on previously published methods [57]. Nine WT zebrafish and nine *elovl6^−/−^* zebrafish (each kind of zebrafish three to a biologic sample) were randomly selected and their livers were taken for analysis. The samples were grinded with liquid nitrogen and transferred to 5 mL centrifuge tubes. Then four volumes of lysis buffer (8M urea, 1% protease inhibitor cocktail, 1% phosphatase inhibitor cocktail (for protein post-translational modification (PTM) experiment)) were added to the powder, and the sample mixture was sonicated for three times on ice with a high-intensity ultrasonic processor (Scientz Biotechnology, Ningbo, China). The remaining fragments were centrifuged at 12,000× g, 4 °C for 10 min. After that, the supernatant was collected and the protein concentration was measured with the bicinchoninic acid (BCA) kit (Beyotime Biotechnology, Shanghai, China) and details were carried out according to the manufacturer’s instructions. The protein solution was reduced with 5-mM dithiothreitol (DTT) for 30 min at 56 °C and alkylated with 11-mM iodoacetamide (IAA) for 15 min at room temperature in the dark. The protein samples were diluted with 100-mM tetraethylammonium bromide (TEAB) to reduce the urea concentration <2M. Trypsin (Promega Corporation, Madison, USA) was added at 1:50 trypsin-to-protein mass ratio for the first step of digestion overnight and 1:100 trypsin-to-protein mass ratio for the second step of digestion for 4 h.

#### 4.7.2. TMT Labeling

After digestion with trypsin, the peptide was desalted by the Strata X C18 SPE column (Phenomenex), followed by vacuum-drying. The peptide was dissolved with 0.5 M TEAB, and TMT labeling was performed according to the manufacturer’s instructions of the TMT kit (Thermo Fisher Scientific, Waltham, MA, USA). The brief instructions were as follows. TMT reagent (one unit) was thawed and dissolved in acetonitrile (Fisher Chemical, Waltham, MA, USA). The peptide mixtures were incubated for 2 h at room temperature. After that, the mixtures were pooled, desalted and dried by vacuum centrifugation.

#### 4.7.3. High Performance Liquid Chromatography (HPLC) Fractionation and Immobilized Metal Affinity Chromatography (IMAC) Enrichment

The tryptic peptides were fractionated into fractions by high pH reverse-phase HPLC using Agilent 300 Extend C18 column (5-μm particles, 250 mm × 4.6 mm) so that the sample complexity was reduced. A gradient of 8% to 32% acetonitrile (pH = 9.0) was used for peptide separation over 60 min into 60 fractions. Then, the peptides were combined into the final 9 fractions (or 6 fractions for PTM experiment) and dried by vacuum centrifuging before LC-MS/MS analysis.

Peptide mixtures were first dissolved in the loading buffer (50% acetonitrile/6% trifluoroacetic acid) and incubated with IMAC microspheres suspension on a rotary shaker with gentle shaking. After incubation, the phosphopeptides in the IMAC microspheres were collected by centrifugation. Then the IMAC microspheres were washed with 50% acetonitrile/6% trifluoroacetic acid and 30% acetonitrile/0.1% trifluoroacetic acid to remove nonspecifically adsorbed peptides for three times, in turn. Finally, in order to elute the enriched phosphopeptides from the IMAC microspheres, the phosphopeptides were eluted with elution buffer containing 10% NH_4_OH by vibration and vacuum-dried. After that, the supernatant was desalted according to the instructions and then collected for LC-MS/MS analysis.

#### 4.7.4. LC-MS/MS Analysis

The tryptic peptides were dissolved in 0.1% solvent A (0.1% (*v*/*v*) formic acid in water) loaded onto a reversed-phase analytical column (15-cm length, 75 μm i.d.) and separated using the EASY-nLC 1000 ultra-performance liquid chromatography (UPLC) system. Mobile phase A contained 0.1% formic acid and 2% acetonitrile, and mobile-phase B contained 0.1% formic acid and 90% acetonitrile. Liquid gradient was setting as following: 0–40 min, an increase from 4–22% solvent B; 40–52 min, 22–32% B; 52–56 min, 32–80% B; 56–60 min, holding at 80% solvent B. Flow rate maintained at 450 nL/min.

The peptides were separated by the UPLC system and subjected to NSI source for ionization, and then analyzed by tandem mass spectrometry in Q Exactive^TM^ Plus (Thermo Fisher Scientific, Waltham, MA, USA). The electrospray voltage was set as 2.0 kV, and the peptide precursor and its secondary fragments were detected and analyzed using high-resolution orbitrap. The primary mass spectrometer scan range was set to 350–1800 m/z at the scan resolution of 70,000. The secondary mass spectrometry scan range was fixed at 100 m/z and the scan resolution was set to 35,000. The data acquisition mode used a data dependent acquisition (DDA) program. To improve the effective utilization of the mass spectrum, the automatic gain control (AGC) was set at 5E4 and then the signal threshold was set to 20,000 ions/s and the maximum injection time was set to 200 ms with 30.0 s dynamic exclusion.

#### 4.7.5. Database Search

The LC-MS/MS data were processed using Maxquant search engine (v.1.5.2.8). Retrieval parameter settings: The database was UniProt *Danio rerio* (44,132 sequences). We added reverse decoy database to calculate FDR caused by random matches. Trypsin/P was specified as the cleavage enzyme. The number of missing cleavages was allowing up to two. The minimum peptide length was set to 7 amino acid residues. The maximum number of peptide modifications was set to 5. The mass tolerances for the primary precursor ions in the first search and main search were set as 20 ppm and 5 ppm. The mass tolerances for fragment ions were set as 0.02 Da. Carbamidomethyl on cysteine was specified as fixed modification. Oxidation on Met, N-terminal acetylation of protein, desamidization of asparagine and phosphorylation of serine, threonine and tyrosine were specified as variable modifications. The quantitative method was set to TMT-10plex and the FDR was adjusted to <1%.

#### 4.7.6. Subcellular Localization

We used WoLF PSORT (http://wolfpsort.seq.cbrc.jp/), a subcellular localization predication soft to predict subcellular localization in this study.

#### 4.7.7. Motif Analysis

The software MoMo (http://meme-suite.org/tools/momo), a motif-x algorithm was used to analyze the model of sequences, in which amino acids constituted at specific positions with modify-13-mers. The identified phosphosites were 6 upstream and downstream; the background database parameters were set as all the database of protein sequences. The minimum number of occurrences was set to 20. The statistical test *p*-value was set to less than 0.000001. Emulate original motif-x was ticked: other parameters were with default.

#### 4.7.8. Functional Analysis

GO annotations of proteomic and phosphoproteomic data were performed based on the UniProt-GOA database (http://www.ebi.ac.uk/GOA/). First, the identified protein ID was converted into UniProt ID, and then UniProt ID was mapping to GO ID. The information was obtained from the UniProt-GOA database according to GO ID. If some identified proteins were not annotated by the UniProt-GOA database, a software based on the protein sequence alignment method, InterProScan (http://www.ebi.ac.uk/interpro/), would be used to annotate the protein’s GO function. GO annotation of proteomics was classified into three categories: BP, CC and MF.

KEGG database (https://www.genome.jp/kegg/) was used for annotation analysis of protein pathway. First, KEGG automatic annotation server (KAAS) (https://www.genome.jp/tools/kaas/) was used to annotate protein’s KEGG database description. Then the annotation result was mapping on the KEGG pathway database by KEGG mapper (https://www.genome.jp/kegg/mapper.html). These pathways were classified into hierarchical categories according to the KEGG website.

A two-tailed Fisher’s exact test was utilized to test the DEP and DEPP against all identified proteins. *p*-value <0.05 was considered significant.

#### 4.7.9. Protein-Protein Interaction Network

DEP or DEPP database accessions or sequences were searched against the STRING database version 11.0 (https://string-db.org/) for protein-protein interaction network.

### 4.8. Data Availability

All transcriptome data are available in the NCBI Sequence Read Archive (SRA) database under accession SRP251016. All proteome and phosphoproteome data are available in the PRoteomics IDEntifications Database (PRIDE) under accession PXD017702.

### 4.9. Statistical Analysis

Data were expressed as mean ± SD (n was indicated in each case). All data were subjected to t-test using SPSS 19.0 (Michigan Avenue, Chicago, IL, USA). *p* < 0.05 was considered statistically significant.

## Supplementary Materials

Supplementary materials can be found at https://www.mdpi.com/1422-0067/21/8/2860/s1. Figure S1. Quantitative PCR test of *elovl6* expression levels and the experiment flow chart of omic analysis. (A). Relative mRNA levels of *elovl6* in livers of wild type zebrafish (WT) and *elovl6^−/−^* zebrafish (*n* = 3). (B). The workflow for hepatic transcriptomic, proteomic and phosphoproteomic analyses of WT and *elovl6^−/−^* zebrafish. Lives of 18 male wild type zebrafish (WT) and 18 male *elovl6^−/−^* zebrafish were used to perform RNA-Seq, TMT labeling-based quantitative proteomic and phosphoproteomic analyses. A bioinformatics analysis of differentially expressed genes (DEG), differentially expressed proteins (DEP) and differentially expressed phosphoproteins (DEPP) with sites was carried out. *elovl6*, elongation of very long chain fatty acids protein 6; TMT, tandem mass tag; LC-MS/MS, liquid chromatography-tandem mass spectrometry. Figure S2. Gene expression levels revealed by quantitative PCR (right side) and RNA-seq (left side). *acsbg2*, Acyl-CoA synthetase bubblegum family member 2; *fasn*, fatty acid synthase; *gck*, phosphotransferase; *pck2*, phosphoenolpyruvate carboxykinase 2 (mitochondrial); *slc27a1b*, solute carrier family 27 member 1b; *gys2*, glycogen synthase 2 (liver); *hkdc1*, hexokinase domain-containing 1; *lpl*, lipoprotein lipase; *ucp1*, mitochondrial uncoupling protein 1. Figure S3. Length distribution of peptides identified by mass spectrometry. A and B. Most of the peptides were 7–20 amino acids in length by proteomic (A) and phosphoproteomic (B) analysis, which conformed to the general rules based on trypsin hydrolysis and high energy collision-induced dissociation (HCD) fragmentation. Figure S4. KEGG enrichment clustering heat map of proteins/genes under different regulatory relationships in transcriptome and proteome. KEGG, kyoto encyclopedia of genes and genomes; Pr, proteome; Tr, transcriptome. Figure S5. The basic statistical data of phosphoproteomics. In the identification of modifications, a total of 79,962 secondary spectra were obtained by the mass spectrometry. After searching the database of protein secondary data for mass spectrometry, the number of available spectra was 15,311. 7887 peptides, 6714 phosphorylated peptides, and 8727 phosphosites on 3199 phosphoproteins were identified. Figure S6. The number of modification sites corresponding to each protein. Figure S7. Comprehensive heatmaps for cluster analysis of the enrichment patterns of GO functional categories. (A). Functional enrichment of differentially expressed proteins (DEP); (B). Functional enrichment of differentially expressed phosphoproteins (DEPP). The related functions are brought together using a hierarchical clustering method, and the description of relevant functions of the enrichment is made vertically. Red means strong enrichment degree, and blue weak enrichment degree. GO, gene ontology. Table S1. Whole-body fatty acid compositions of wild type (WT) and *elovl6^−/−^* zebrafish. Table S2. The differentially expressed genes (DEG) (fold change>2 and p-adjust <0.005) associated with insulin signaling pathway, PPAR signaling pathway and glycolysis / gluconeogenesis. Table S3. Summary of transcriptomic, proteomic and phosphoproteomic data. Table S4. The differentially expressed proteins (DEP) (fold change >1.5, p-value<0.05) associated with insulin signaling pathway, PPAR signaling pathway and glycolysis/gluconeogenesis. Table S5. The differentially expressed phosphoproteins (DEPP) (fold change >1.5, p-value<0.05) associated with insulin signaling pathway, glycolysis / gluconeogenesis and lipid metabolism. Table S6. The feature sequences of modified sites and its enrichment statistics. Table S7. List of the differentially expressed kinases associated with insulin signaling pathway, mTOR signaling pathway, MAPK signaling pathway, GnRH signaling pathway and apelin signaling pathway in phosphoproteomic analysis, with fold-change > 1.5 and *p*-value < 0.05. Table S8. Primers used for qPCR analysis.

## Abbreviations

*acsbg2*	acyl-CoA synthetase bubblegum family member 2
*acsl1*	acyl-CoA synthetase long chain family member 1
*acsl5*	acyl-CoA synthetase long chain family member 5
AGC	automatic gain control
*akt2l*	V-akt murine thymoma viral oncogene homolog 2, -like
BCA	bicinchoninic acid
BHT	butylated hydroxytoluene
BP	biologic process
CaMK II	calcium/calmodulin kinase II
CC	cellular compartment
CDK	cyclin-dependent kinase
ChREBP	carbohydrate response element binding protein
CKII	casein kinase II
DDA	data dependent acquisition
DEG	differentially expressed genes
DEP	differentially expressed proteins
DEPP	differentially expressed phosphoproteins
DTT	dithiothreitol
*elovl6*	elongation of very long chain fatty acids protein 6
FAME	fatty acid methyl esters
*fasn*	fatty acid synthase
FDR	false discovery rate
FPKM	fragments per kilobases per million reads
*gck*	phosphotransferase
G-CK	Golgi casein kinase
GnRH	gonadotropin-releasing hormone
GO	gene ontology
*gys2*	glycogen synthase 2 (liver)
HCD	high energy collision-induced dissociation
HF-HS	high-fat, high-sucrose
*hkdc1*	hexokinase domain-containing 1
HPLC	high performance liquid chromatography
HT	high-throughput-screening technology
IAA	iodoacetamide
IMAC	immobilized metal affinity chromatography
KEGG	Kyoto encyclopedia of genes and genomes
KO	knock-out
LC-MS/MS	liquid chromatography-tandem mass spectrometry
*ldlr*	low-density lipoprotein receptor
*ldhbb*	L-lactate dehydrogenase B-B chain
*lpl*	lipoprotein lipase
MARS	MA-plot-based method with random sampling
MAPK	mitogen-activated protein kinase
Map2k6	mitogen-activated protein kinase 6
MF	molecular function
mTOR	mammalian target of rapamycin
NAFLD	non-alcoholic fatty liver disease
NCBI	national center for biotechnology information search database
PCA	principal component analysis
*pck1*	phosphoenolpyruvate carboxykinase 1
*pck2*	phosphoenolpyruvate carboxykinase 2 (mitochondrial)
PKCε	protein kinase C epsilon type
PPAR	peroxisome proliferators-activated receptor
Prkab1b	protein kinase, AMP-activated, beta 1 non-catalytic subunit, b
PPI	protein-protein interactions
PTM	post-translational modification
*rps6kb*	ribosomal protein S6 kinase
*scd1*	stearoyl-CoA desaturase 1
*slc27a1b*	solute carrier family 27-member 1b
*srebp-1*	sterol regulatory element-binding protein 1
T2D	type 2 diabetes
TEAB	tetraethylammonium bromide
TMT	tandem mass tag
*ucp1*	mitochondrial uncoupling protein 1
*ucp2*	mitochondrial uncoupling protein 2
UPLC	ultra-performance liquid chromatography
