# Supplementary material for: Quantitative Phosphoproteomic Analysis Reveals the Regulatory Networks of Elovl6 on Lipid and Glucose Metabolism in Zebrafish
Source: Int J Mol Sci. 2020 Apr 19;21(8):2860. doi: 10.3390/ijms21082860 (PMC7215441; doi:10.3390/ijms21082860)
Supplement: Supplementary file 1 [file ijms-21-02860-s001.pdf]

A.

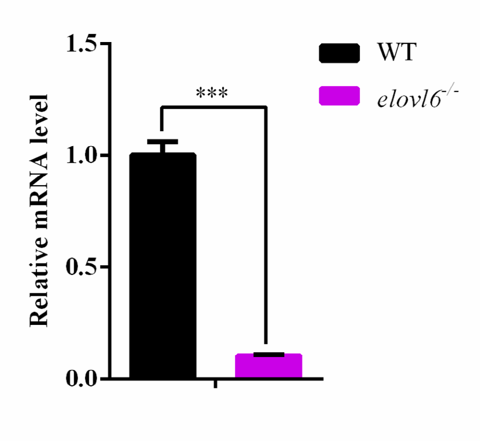

B.

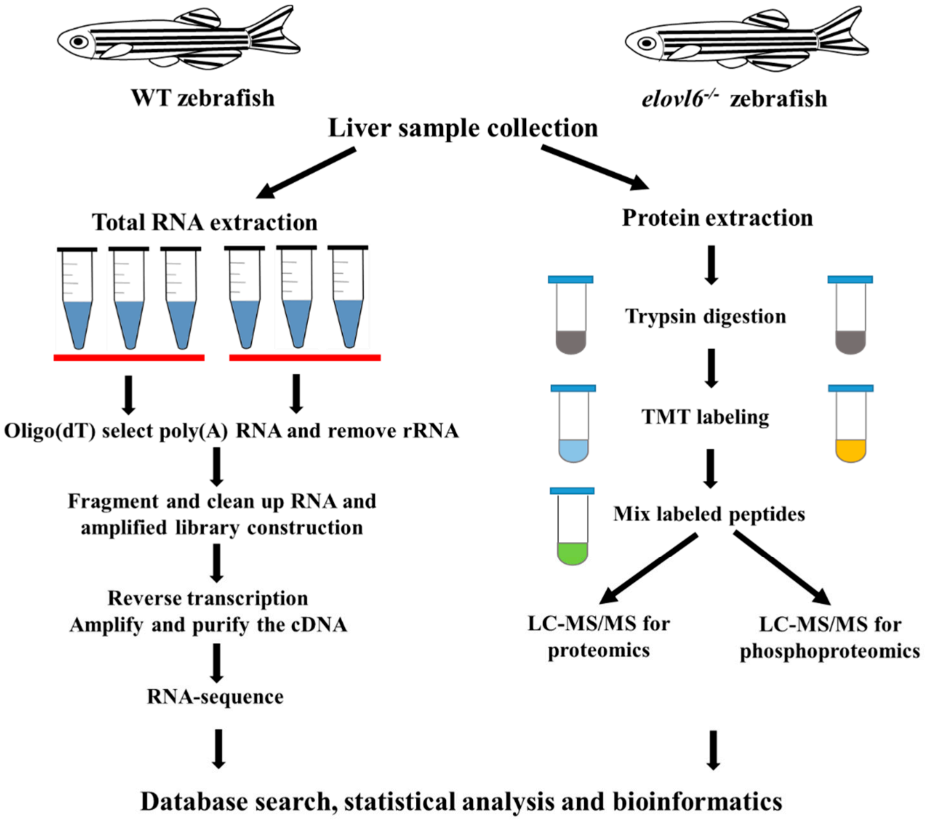

Figure S1. Quantitative PCR test of *elovl6* expression levels and the experiment flow chart of omic analysis. **A.** Relative mRNA levels of *elovl6* in livers of wild type zebrafish (WT) and *elovl6*$^{-/-}$ zebrafish (n = 3). **B.** The workflow for hepatic transcriptomic, proteomic and phosphoproteomic analyses of WT and *elovl6*$^{-/-}$ zebrafish. Lives of 18 male wild type zebrafish (WT) and 18 male *elovl6*$^{-/-}$ zebrafish were used to perform RNA-Seq, TMT labeling-based quantitative proteomic and phosphoproteomic analyses. A bioinformatics analysis of differentially expressed genes (DEG), differentially expressed proteins (DEP) and differentially expressed phosphoproteins (DEPP) with sites was carried out.

elovl6, elongation of very long chain fatty acids protein 6; TMT, tandem mass tag; LC-MS/MS, liquid chromatography-tandem mass spectrometry.

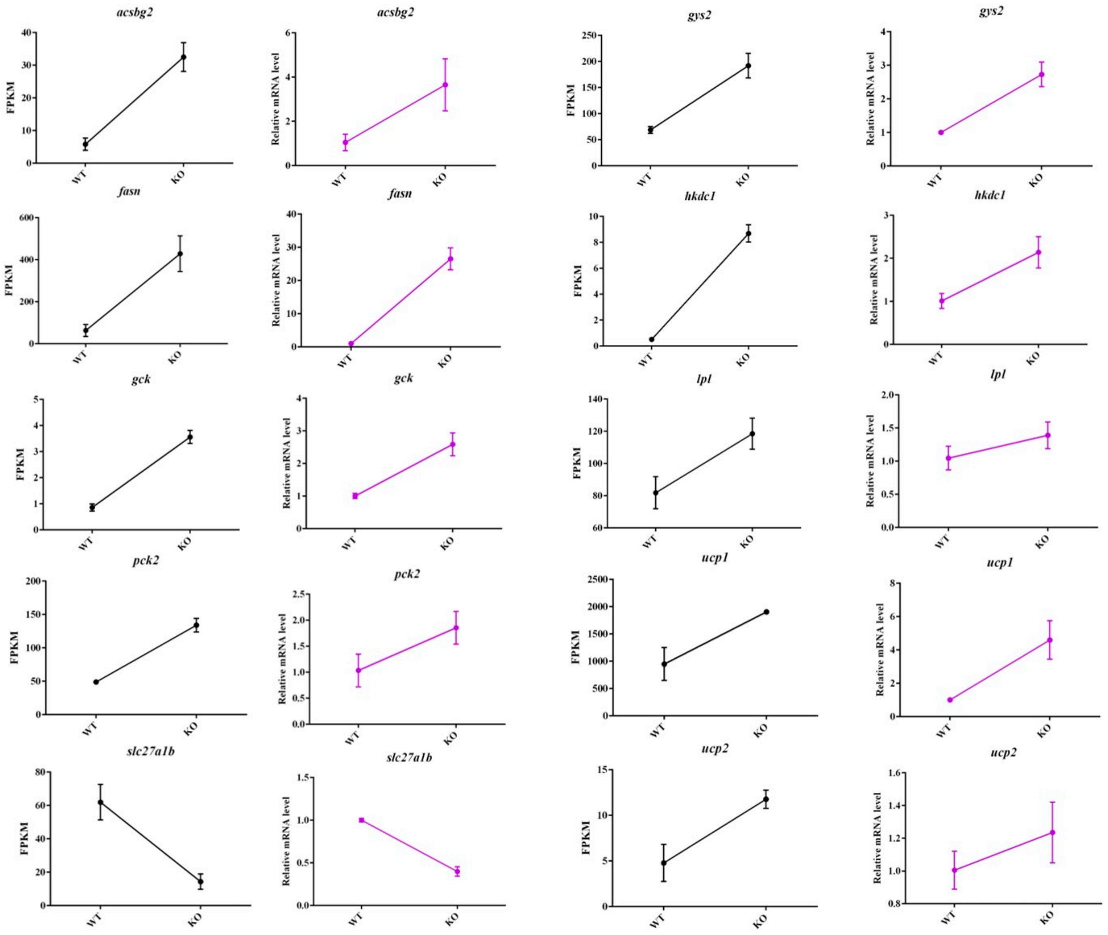

Figure S2. Gene expression levels revealed by quantitative PCR (right side) and RNA-seq (left side). *acsbg2*, Acyl-CoA synthetase bubblegum family member 2; *fasn*, fatty acid synthase; *gck*, phosphotransferase; *pck2*, phosphoenolpyruvate carboxykinase 2 (mitochondrial); *slc27a1b*, solute carrier family 27 member 1b; *gys2*, glycogen synthase 2 (liver); *hkdc1*, hexokinase domain-containing 1; *lpl*, lipoprotein lipase; *ucp1*, mitochondrial uncoupling protein 1.

A.

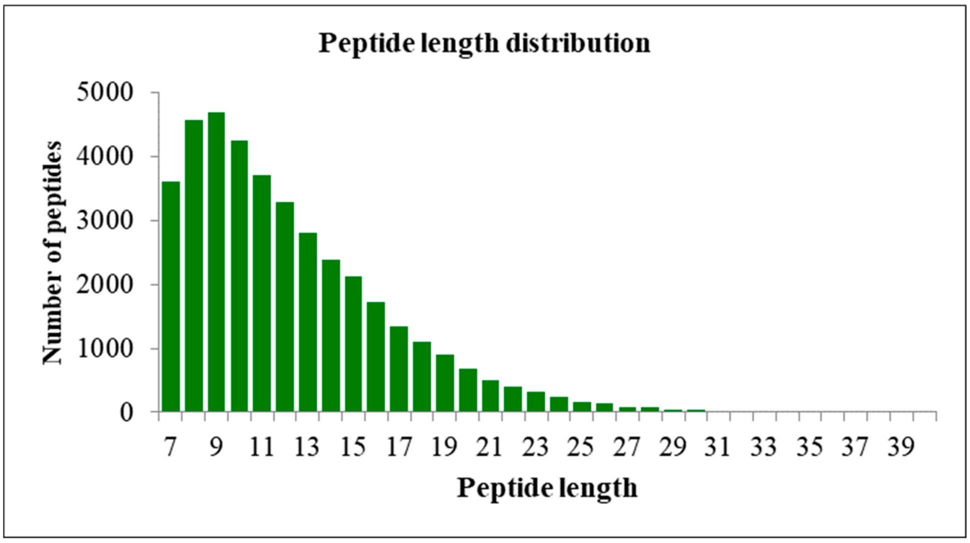

B.

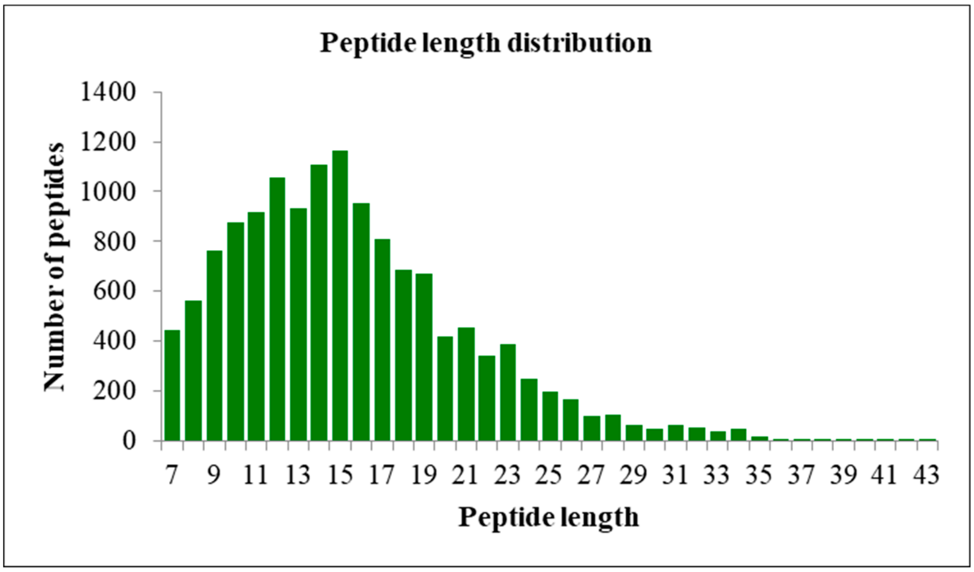

Figure S3. Length distribution of peptides identified by mass spectrometry**.** A and B. Most of the peptides were 7-20 amino acids in length by proteomic (**A**) and phosphoproteomic (**B**) analysis, which conformed to the general rules based on trypsin hydrolysis and high energy collision-induced dissociation (HCD) fragmentation.

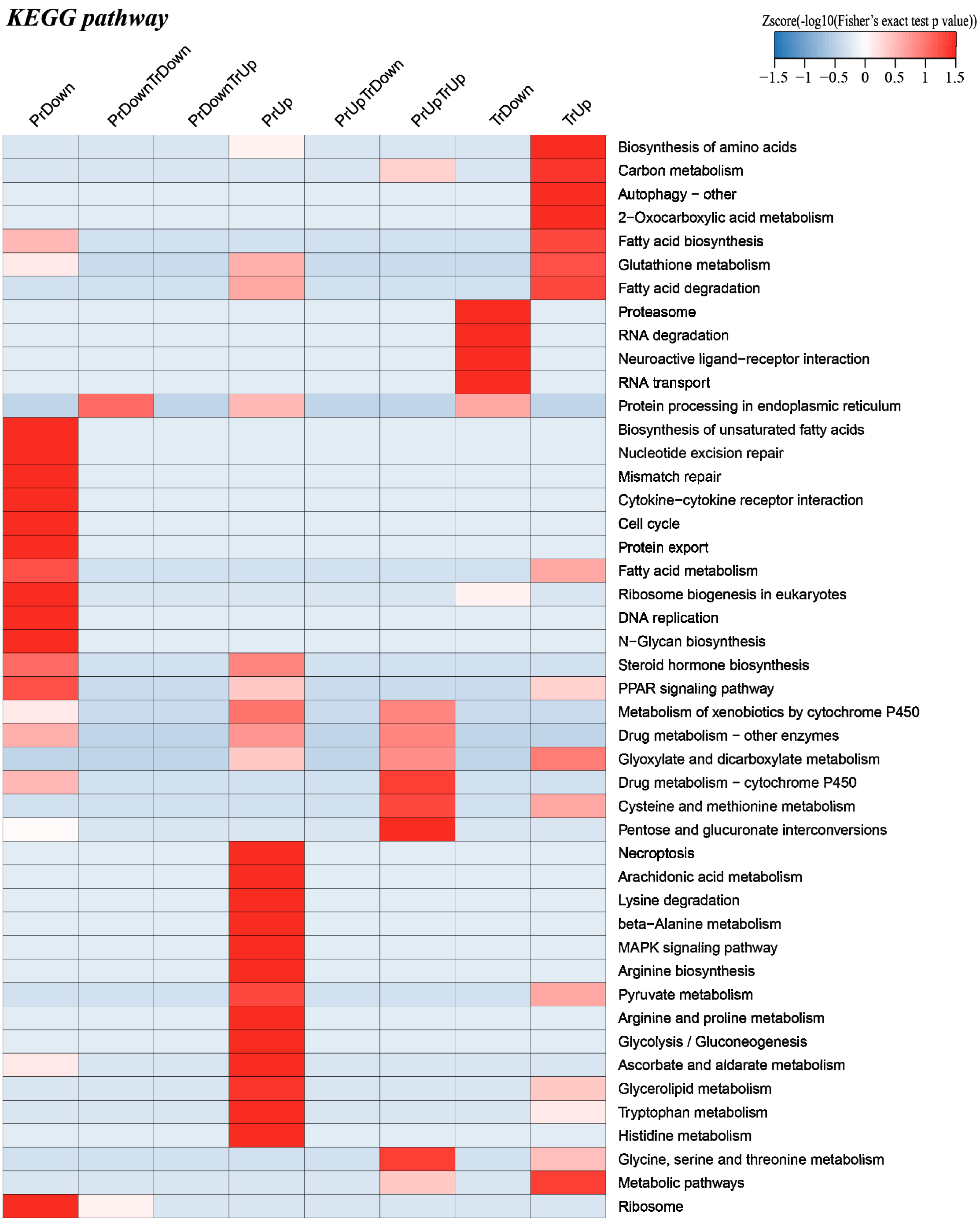

Figure S4. KEGG enrichment clustering heat map of proteins/genes under different regulatory relationships in transcriptome and proteome. KEGG, kyoto encyclopedia of genes and genomes; Pr, proteome; Tr, transcriptome.

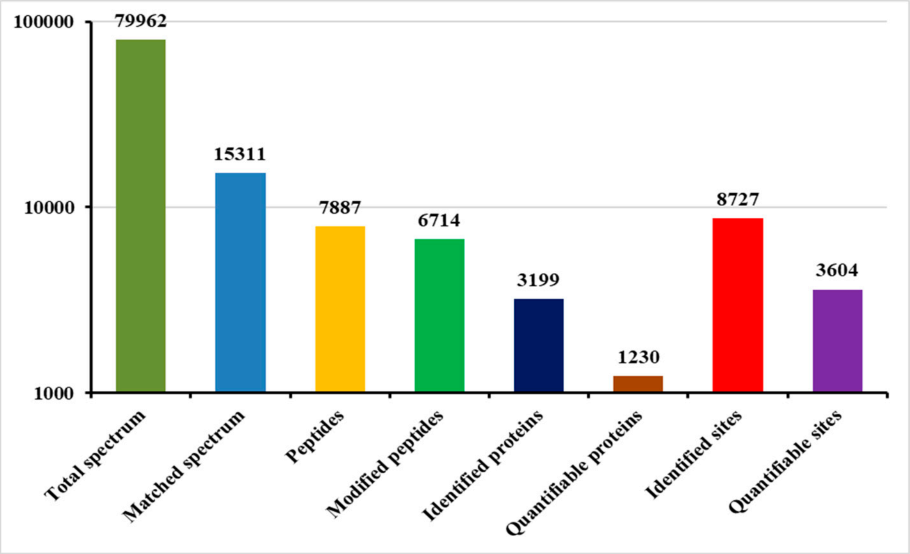

Figure S5. The basic statistical data of phosphoproteomics. In the identification of modifications, a total of 79,962 secondary spectra were obtained by the mass spectrometry. After searching the database of protein secondary data for mass spectrometry, the number of available spectra was 15,311. 7,887 peptides, 6,714 phosphorylated peptides, and 8,727 phosphosites on 3,199 phosphoproteins were identified.

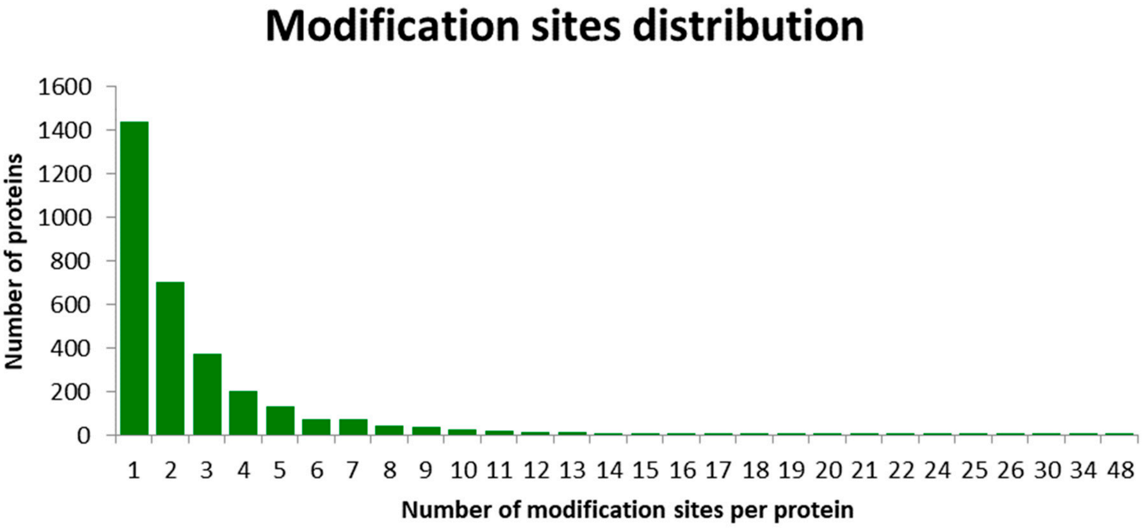

Figure S6. The number of modification sites corresponding to each protein

A.

B.

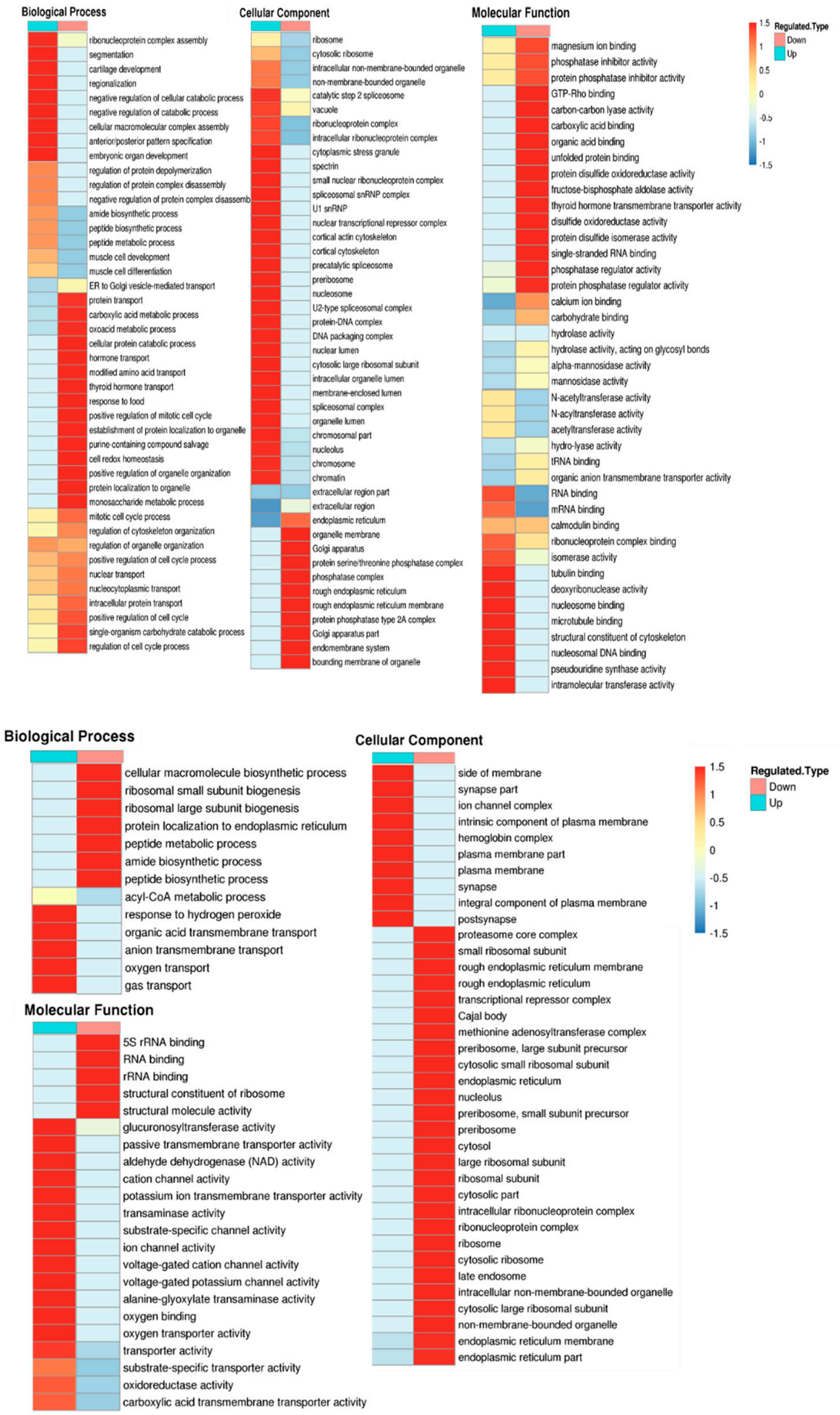

Figure S7. Comprehensive heatmaps for cluster analysis of the enrichment patterns of GO functional categories. (**A**). Functional enrichment of differentially expressed proteins (DEP); (**B**). Functional enrichment of differentially expressed phosphoproteins (DEPP). The related functions are brought together using a hierarchical clustering method, and the description of relevant functions of the enrichment is made vertically. Red means strong enrichment degree, and blue weak enrichment degree. GO, gene ontology.

Table S1. Whole-body fatty acid compositions of wild type (WT) and *elovl6*$^{-/-}$ zebrafish

Fatty acids	WT zebrafish	*elovl6*$^{-/-}$ zebrafish	*p*-value	Significance
14:0	0.7±0.1	1.3±0.1	0.000466	***
16:0	20.1±0.4	29.3±0.4	1.5052E-7	***
18:0	7.1±0.1	4.6±0.1	2.3093E-8	***
20:0	0.5±0.0	1.1±0.1	0.000119	***
22:0	0.0±0.0	0.0±0.0	0.373901	ns
16:ln-7	3.4±0.1	5.6±0.1	0.000003	***
18:ln-9/ln-7	31.0±0.5	23.2±0.3	8.2148E-7	***
20:ln-9	0.7±0.0	0.8±0.0	0.069360	ns
22:ln-11	0.2±0.1	0.4±0.0	0.153939	ns
18:2n-6	23.2±0.9	18.8±0.3	0.006060	**
18:3n-6	0.7±0.1	0.4±0.0	0.000294	***
20:2n-6	1.4±0.1	1.1±0.0	0.005278	**
20:4n-6	2.7±0.1	3.2±0.1	0.013852	*
22:4n-6	1.0±0.1	0.5±0.0	0.006464	**
18:3n-3	3.0±0.1	3.0±0.2	0.901461	ns
18:4n-3	0.3±0.0	0.5±0.1	0.082050	ns
20:4n-3	0.2±0.0	0.4±0.0	0.000655	***
20:5n-3	1.0±0.1	1.6±0.2	0.020993	*
22:5n-3	0.1±0.0	0.4±0.1	0.004276	*
22:6n-3	2.6±0.1	4.0±0.2	0.000344	***

Results are represented as means ± SD. *p<0.05, **p<0.01, ***p<0.001. ns, no significant difference. *elovl6*, elongation of very long chain fatty acids protein 6.

Table S2. The differentially expressed genes (DEG) (fold change>2 and p-adjust <0.005) associated with insulin signaling pathway, PPAR signaling pathway and glycolysis / gluconeogenesis

Gene name	Description	Fold-change	P-adjust
**Insulin signaling pathway**			
eif4ea	Eukaryotic translation initiation factor 4ea	11.992	0.0002737
prkag3b	Protein kinase, AMP-activated, gamma 3b non-catalytic subunit	10.913	0.002849
fasn	Fatty acid synthase	6.802	1.977E-07
mknk2a	MAPK-interacting serine/threonine kinase 2a	5.109	2.559E-07
mknk2b	MAPK-interacting serine/threonine kinase 2b	4.26	8.153E-09
calm2a	Calmodulin	4.208	0.0007881
pik3r3a	Phosphoinositide-3-kinase, regulatory subunit 3a	3.55	0.002044
pygl	Alpha-1,4 glucan phosphorylase	2.778	0.000529
mknk1	MAPK-interacting serine/threonine kinase 1	0.216	0.0002903
socs3b	Suppressor of cytokine-signaling 3b	0.207	4.228E-06
ppp1r3ca	Protein phosphatase 1 regulatory subunit 3C	0.014	5.994E-12
**PPAR siganling pathway**			
slc27a6	Solute carrier family 27 member 6	8.236	3.403E-07
ubb	Ubiquitin B	5.51	8.261E-07
apoa1a	Apolipoprotein A-I	5.355	0.0002183
acsbg2	Acyl-CoA synthetase bubblegum family member 2	4.228	0.0009148
zgc:172295	zgc:172295	2.963	0.0003046
slc27a1b	Solute carrier family 27 member 1b	0.19	3.269E-06
cyp7a1	Cholesterol 7-alpha-monooxygenase	0.075	6.436E-09
**Glycolysis / Gluconeogenesis**			
adh5	S-(hydroxymethyl)glutathione dehydrogenase	5.333	0.0003942
ldhbb	L-lactate dehydrogenase B-B chain	3.494	0.00003766
minpp1a	Multiple inositol-polyphosphate phosphatase 1a	0.271	0.002411

PPAR, peroxisome proliferators-activated receptor.

Table S3. Summary of transcriptomic, proteomic and phosphoproteomic data

**Category**	***elvol6*$^{-/-}$ zebrafish vs WT zebrafish**
Quantifiable transcripts/differentially expressed genes (DEG)	47251/734
Up/down-regulated DEG	335/399
Quantifiable proteins/differentially expressed proteins (DEP)	5525/559
Up/down-regulated DEP	242/317
Quantifiable phosphoproteins /differentially expressed phosphoproteins (DEPP)	1230/680
Up/down-regulated DEPP	224/456
Quantifiable phosphopeptides /differentially expressed phosphosites	3604/1054
Up/down-regulated phosphosites	289/765

elovl6, elongation of very long chain fatty acids protein 6; WT, wild type.

Table S4. The differentially expressed proteins (DEP) (fold change >1.5, p-value<0.05) associated with insulin signaling pathway, PPAR signaling pathway and glycolysis/gluconeogenesis

Protein name	Protein description	Fold-change	*p*-value
**Insulin signaling pathway**			
Pygma	Alpha-1,4 glucan phosphorylase	2.628	1.70652E-05
Ins	Insulin	1.876	4.8624E-06
Fbp1b	Fructose-1,6-bisphosphatase 1b	1.501	3.2108E-06
Mapk1	Mitogen-activated protein kinase	0.654	0.0035626
Pck1	Phosphoenolpyruvate carboxykinase 1	0.536	0.000016777
**PPAR signaling pathway**			
Slc27a6	Solute carrier family 27, member 6	2.677	5.986E-07
Fabp3	Fatty acid-binding protein 3, muscle and heart	1.844	0.000136128
Cyp27a1.2	Cytochrome P450, family 27, subfamily A, polypeptide 1, gene 2	1.702	3.3325E-07
Zgc:101540	Zgc:101540 protein	1.535	0.000037168
Plin1	Perilipin 1	0.641	0.00040157
Scd	Stearoyl-CoA desaturase (delta-9-desaturase)	0.614	0.031942
Apoa1	Apolipoprotein A-I	0.556	2.2354E-07
Pck1	Phosphoenolpyruvate carboxykinase 1	0.536	0.000016777
Acsl1b	Acyl-CoA synthetase long chain family member 1b	0.521	2.1661E-06
Acsl5	Acyl-CoA synthetase long chain family member 5	0.463	0.0092213
**Glycolysis / Gluconeogenesis**			
Pkmb	Pyruvate kinase	2.084	0.00032295
Slc2a5	Solute carrier family 2, member 5	1.976	0.00065766
Ldhbb	L-lactate dehydrogenase B-B chain	1.822	2.9588E-06
Aldh2.1	Aldehyde dehydrogenase 2 family, tandem duplicate 1	1.708	0.0003216
Akr1a1a	Alcohol dehydrogenase [NADP(+)] A	1.653	0.00032328
Aldh9a1b	Aldehyde dehydrogenase family 9 member A1-B	1.519	0.000057412
Gyg2	Glycogenin 2	1.507	0.0023806
Fbp1b	Fructose-1,6-bisphosphatase 1b	1.501	3.2108E-06
Pck1	Phosphoenolpyruvate carboxykinase 1	0.536	0.000016777
**Fatty acid metabolism**			
Hsd17b12b	Very-long-chain 3-oxoacyl-CoA reductase-B	0.662	7.6247E-05
Scd	Stearoyl-CoA desaturase (delta-9-desaturase)	0.614	0.031942
Elovl5	Elongation of very long chain fatty acids protein 5	0.596	0.0032442
Acsl1b	Acyl-CoA synthetase long chain family member 1b	0.521	2.17E-06
Acsl5	Acyl-CoA synthetase long chain family member 5	0.463	0.0092213

PPAR, peroxisome proliferators-activated receptor.

Table S5. The differentially expressed phosphoproteins (DEPP) (fold change >1.5, p-value<0.05) associated with insulin signaling pathway, glycolysis / gluconeogenesis and lipid metabolism

Protein name	Protein description	phospho sites	Fold-change
**Insulin signaling pathway**			
Hdac8	Phosphorylase b kinase regulatory subunit	S^{1024}	2.409
Akt2l	V-akt murine thymoma viral oncogene homolog 2,-like	T^{449}	1.786
Prkab1b	Protein kinase, AMP-activated, beta 1 non-catalytic subunit, b	S^{34}	1.747
Phka2	Phosphorylase b kinase regulatory subunit	S^{715}	1.566
Rps6kb1a	Ribosomal protein S6 kinase	S^{421}	0.641
Eif4ebp1	Eukaryotic translation initiation factor 4E-binding protein 1	T^{34}	0.636
Crkl	V-crk avian sarcoma virus CT10 oncogene homolog-like	S^{138}	0.625
Crkl	V-crk avian sarcoma virus CT10 oncogene homolog-like	S^{304}	0.595
Eif4ebp1	Eukaryotic translation initiation factor 4E-binding protein 1	T^{67}	0.587
Rps6	40S ribosomal protein S6	S^{240}	0.504
Phkb	Phosphorylase b kinase regulatory subunit	S^{914}	0.474
Rps6kb1b	Ribosomal protein S6 kinase	S^{401}	0.472
Rps6	40S ribosomal protein S6	S^{244}	0.447
Rps6	40S ribosomal protein S6	S^{236}	0.439
Pck2	Phosphoenolpyruvate carboxykinase 2 (mitochondrial)	S^{329}	0.374
Rps6	40S ribosomal protein S6	S^{235}	0.371
Rps6kb1b	Ribosomal protein S6 kinase	S^{421}	0.276
**Glycolysis/Gluconeogenesis**			
Pgm1	Phosphoglucomutase 1	S^{504}	3.929
Pklr	Pyruvate kinase	S^{441}	1.655
Aldoab	Fructose-bisphosphate aldolase	S^{46}	0.646
Pklr	Pyruvate kinase	S^{17}	0.634
Aldob	Fructose-bisphosphate aldolase B	S^{353}	0.619
Gapdh-2	Glyceraldehyde-3-phosphate dehydrogenase	S^{254}	0.607
Aldob	Fructose-bisphosphate aldolase B	T^{39}	0.554
Pck2	Phosphoenolpyruvate carboxykinase 2 (mitochondrial)	S^{329}	0.374
**Lipid metabolism related protein**			
Apc	Adenomatous polyposis coli	S^{1010}	7.703
Osbpl3a	Oxysterol-binding protein	S^{369}	2.914
Pnpla7b	Patatin-like phospholipase domain-containing 7b	S^{299}	1.604
Pi4kb	Phosphatidylinositol 4-kinase beta	S^{447}	1.524
Pi4k2a	Phosphatidylinositol 4-kinase type 2-alpha	S^{430}	0.656
Lpin1	Lipin 1	S^{260}	0.586
Pcyt1aa	Phosphate cytidylyltransferase 1, choline, alpha a	S^{326}	0.58
Hmgcs1	3-hydroxy-3-methylglutaryl coenzyme A synthase	S^{4}	0.575
Osbpl3a	Oxysterol-binding protein	S^{777}	0.559
Pi4kb	Phosphatidylinositol 4-kinase beta	S^{327}	0.538
Vtg6	Vitellogenin 6	S^{1146}	0.521
Vtg6	Vitellogenin 6	S^{1159}	0.52
Pcyt1aa	Phosphate cytidylyltransferase 1, choline, alpha a	S^{366}	0.504
Vtg6	Vitellogenin 6	T^{1151}	0.452
Vtg6	Vitellogenin 6	S^{1155}	0.448
Vtg6	Vitellogenin 6	S^{1152}	0.415
Vtg6	Vitellogenin 6	S^{956}	0.397
Vtg2	Vitellogenin 2	S^{949}	0.386
Vtg6	Vitellogenin 6	S^{1153}	0.307
Apc	Adenomatous polyposis coli	S^{2462}	0.225
Chkb	Choline kinase beta	S^{89}	0.198

Table S6. The feature sequences of modified sites and its enrichment statistics

Motif	Motif Score	Fold Increase	Motif	Motif Score	Fold Increase
xxxRxx_S_Pxxxxx	32.00	16.2	xxxKRx_S_xxxxxx	30.68	8.3
xxxxxx_S_PxxxRx	32.00	12.9	Rxxxxx_S_xDxxxx	23.69	7.3
xxxGRx_S_Pxxxxx	39.24	31.7	xxxxxG_S_xExxxx	23.20	5.7
xxxxxx_S_PRxxxx	31.00	11.1	xxxRxx_S_xxxxxx	16.00	3.7
xxxxxx_S_PxRxxx	30.08	10.5	xxxxxx_S_DxDxxx	29.62	9.2
xxxRRx_S_xxxxxx	32.00	13.6	xxxxxx_S_xEExxx	27.43	6.0
xxxxPx_S_Pxxxxx	29.93	8.8	xxxxRx_S_xxxxxx	16.00	2.7
xxxxRx_S_Pxxxxx	26.50	10.3	xxxxxx_S_xDDxxx	22.81	8.1
xxxRxx_S_xDxxxx	29.51	10.0	RxxSxx_S_xxxxxx	22.84	5.3
xxxxxx_S_PxxxxK	26.38	10.1	xxxxxx_S_xDxxxx	16.00	2.6
xxxRxx_S_xExxxx	30.15	9.2	xxxxxx_S_xExxxx	14.06	2.2
Rxxxxx_S_Pxxxxx	26.86	10.9	xxxxSx_S_xxDxxx	22.18	6.1
xxxxxx_S_DDExxx	45.05	38.5	xxxxxG_S_xxxxxx	14.07	2.1
xRxRTx_S_xxxxxx	33.50	24.8	Rxxxxx_S_xxxxxx	12.28	2.4
xxxxxx_S_PxxxxR	24.34	9.6	xxxxxx_S_xPxxxx	11.77	2.1
xxxRxx_S_xxDxxx	25.88	9.3	xxxxxx_S_xxGxxx	9.72	2.1
xxxxxx_S_DEExxx	42.48	27.9	xxxxxx_S_xxExxx	8.81	2.1
xLxRSx_S_xxxxxx	38.27	16.9	xxxxxD_S_xxxxxx	7.99	2.3
xxRxxx_S_Pxxxxx	23.41	10.1	xxxxxR_S_xxxxxx	6.87	2.2
xxxxxD_S_ExExxx	42.93	20.7	xxxxxx_S_Fxxxxx	7.08	2.5
xxxRxx_S_Lxxxxx	23.43	6.9	xxxxxx_S_xxDxxx	6.10	2.4
xxxxxx_S_PxxRxx	22.61	9.7	xxxxxx_T_PPxxxx	32.00	30.8
xxxxxx_S_DxExxx	32.00	9.4	xxxRxx_T_Pxxxxx	23.01	22.3
xxxxSx_S_Pxxxxx	22.15	6.9	xxxxxx_T_PxxxxR	23.43	23.2
xxRRxx_S_xxxxxx	22.90	7.8	xxxGxx_T_Pxxxxx	22.02	19.7
xxxxxD_S_DxDxxx	43.55	32.1	xxxxxx_T_Pxxxxx	16.00	6.0
xxxxxx_S_xEDxxx	30.68	6.3	xxxRRx_T_xxxxxx	23.19	21.5
xxxxxx_S_xDExxx	32.00	6.8	xxxRSx_T_xxxxxx	24.18	19.7
xxxRxx_S_xPxxxx	23.23	8.5	xxxRxx_T_xxxxxx	9.39	3.5
xxxxxx_S_Pxxxxx	16.00	4.4	xxxxxx_T_xxExxx	8.09	2.9
xxKRxx_S_xxxxxx	22.36	8.9	xxxxxx_T_xxRxxx	6.12	3.1

The minimum number of occurrences was set to 20. $P<0.000001$.

Table S7. List of the differentially expressed kinases associated with insulin signaling pathway, mTOR signaling pathway, MAPK signaling pathway, GnRH signaling pathway and apelin signaling pathway in phosphoproteomic analysis, with fold-change>1.5 and *p*-value<0.05.

**Protein name**	**Protein description**	**phosphosites**	**Fold-change**
**Insulin signaling pathway**			
Hdac8	Phosphorylase b kinase regulatory subunit	S^{1024}	2.409
Akt2l	V-akt murine thymoma viral oncogene homolog 2, -like	T^{449}	1.786
Prkab1b	Protein kinase, AMP-activated, beta 1 non-catalytic subunit, b	S^{34}	1.747
Phka2	Phosphorylase b kinase regulatory subunit	S^{715}	1.566
Rps6kb1a	Ribosomal protein S6 kinase	S^{421}	0.641
Phkb	Phosphorylase b kinase regulatory subunit	S^{914}	0.474
Rps6kb1b	Ribosomal protein S6 kinase	S^{401}	0.472
Rps6kb1b	Ribosomal protein S6 kinase	S^{421}	0.276
**mTOR signaling pathway**			
Akt2l	V-akt murine thymoma viral oncogene homolog 2, -like	T^{449}	1.786
Rps6ka3a	Ribosomal protein S6 kinase	S^{360}	0.663
Rps6ka3a	Ribosomal protein S6 kinase	S^{707}	0.652
Rps6ka3a	Ribosomal protein S6 kinase	S^{218}	0.554
Rps6kb1a	Ribosomal protein S6 kinase	S^{421}	0.641
Rps6kb1b	Ribosomal protein S6 kinase	S^{401}	0.472
Rps6kb1b	Ribosomal protein S6 kinase	S^{421}	0.276
**MAPK signaling pathway**			
Akt2l	V-akt murine thymoma viral oncogene homolog 2, -like	T^{449}	1.786
Rps6ka3a	Ribosomal protein S6 kinase	S^{360}	0.663
Rps6ka3a	Ribosomal protein S6 kinase	S^{707}	0.652
Rps6ka3a	Ribosomal protein S6 kinase	S^{218}	0.554
Map2k6	Dual specificity mitogen-activated protein kinase kinase 6	S^{234}	0.643
Map3k5	Mitogen-activated protein kinase kinase kinase 5	S^{1016}	0.643
Mapk14b	Mitogen-activated protein kinase	T^{181}	0.641
Map4k2	Mitogen-activated protein kinase kinase kinase kinase	S^{481}	0.638
Map4k2	Mitogen-activated protein kinase kinase kinase kinase	S^{459}	0.515
**GnRH signaling pathway**			
Camk2b1	Calcium/calmodulin-dependent protein kinase II beta 1	S^{310}	0.655
Map2k6	Dual specificity mitogen-activated protein kinase kinase 6	S^{234}	0.643
Mapk14b	Mitogen-activated protein kinase	T^{181}	0.641
Prkcdb	Protein kinase C delta type	S^{673}	0.571
**Apelin signaling pathway**			
Akt2l	V-akt murine thymoma viral oncogene homolog 2, -like	T^{449}	1.786
Prkab1b	Protein kinase, AMP-activated, beta 1 non-catalytic subunit, b	S^{34}	1.747
Rps6kb1a	Ribosomal protein S6 kinase	S^{421}	0.641
Rps6kb1b	Ribosomal protein S6 kinase	S^{401}	0.472
Rps6kb1b	Ribosomal protein S6 kinase	S^{421}	0.276

MAPK, mitogen-activated protein kinase; mTOR, mammalian target of rapamycin; GnRH, gonadotropin-releasing hormone.

Table S8 Primers used for qPCR analysis

Genes	Forward primer (5′-3′)	Reverse primer (5′-3′)
gapdh	TCCAGTACGACTCCACCCAT	TGACTCTCTTTGCACCACCC
β-actin	CACCACCACAGCCGAAAGAG	ACCGCAAGATTCCATACCCA
elovl6	AGAGGACCACCAGAGACCT	CTGAAGGCGGCAAGTGTTAA
acsbg2	CACTGCTGCCAAGAGCTTCC	GGATGTTGGCCTGGCAGTTC
fasn	GTGCTGCTACAGGTGCGTTC	TGGACACGAGGACCTGGATG
gck	AAGAGCGAGGCTGGAAGGTG	CACGCATTGCAGCCTGTACC
gys2	CTTTGGCCGTTGGCTGATTG	ACCAGAGAGCCCAGGATGAG
hkdc1	ATGCGGCACATTGACCTGG	GCGGCATCAATTTCACGGTC
lpl	TTGGCGCTCATGTTGCAGG	TGGAGAACCACGGGTGTTG
pck2	AAAGATGTGGCTCGGGTGG	ATAGTGCGTCCTGCCATGC
slc27a1b	TCCTGTGGTTCTGCTCCTGC	ACCACGATGGCAGCTTTAGG
ucp1	TTTCGGGACCATCAGCACC	ATTCGCACCGCCACATTAGG
ucp2	TTCTGTACGCATCGGCCTC	ATGGCTCCAGTCGTACAACC

gapdh, glyceraldehyde-3-phosphate dehydrogenase; *elovl6*, elongation of very long chain fatty acids protein 6; *acsbg2,* Acyl-CoA synthetase bubblegum family member 2; *fasn,* fatty acid synthase; *gck,* phosphotransferase; *pck2*, phosphoenolpyruvate carboxykinase 2 (mitochondrial); *slc27a1b*, solute carrier family 27 member 1b; *gys2,* glycogen synthase 2 (liver); *hkdc1*, hexokinase domain-containing 1; *lpl*, lipoprotein lipase; *ucp1*, mitochondrial uncoupling protein 1.
